# Sleep Pattern, Lifestyle Pattern, and Risks of Overall and 20 Types of Cancers: Findings From the UK Biobank Cohort

**DOI:** 10.3389/ijph.2024.1607726

**Published:** 2025-01-17

**Authors:** Yue-Ze Zhao, Wen-Li Zhang, Kai-Wen Zhang, Yong-Qiao He, Wen-Qiong Xue, Da-Wei Yang, Hua Diao, Ruo-Wen Xiao, Ying Liao, Qiao-Ling Wang, Wei-Hua Jia, Tong-Min Wang

**Affiliations:** ^1^ School of Public Health, Sun Yat-sen University, Guangzhou, China; ^2^ State Key Laboratory of Oncology in South China, Collaborative Innovation Center for Cancer Medicine, Sun Yat-sen University Cancer Center, Guangzhou, China; ^3^ School of Economics, The University of Edinburgh, Edinburgh, United Kingdom

**Keywords:** cancer prevention, UK biobank, healthy sleep pattern, healthy lifestyle pattern, human behaviour

## Abstract

**Objectives:**

Sleep health and other lifestyle behaviours are gaining increasing attention in public health, particularly for cancer prevention, but a comprehensive assessment is lacking.

**Methods:**

The study included 380,042 UK Biobank participants. A healthy sleep score was constructed based on five sleep factors: chronotype, sleep duration, insomnia, snoring, and daytime dozing. A healthy lifestyle score was constructed based on four lifestyle factors: smoking, alcohol consumption, diet and physical activity. The effect of healthy sleep and lifestyle on cancer risk was examined by Cox proportional hazard models.

**Results:**

Both healthy sleep and lifestyle patterns were significantly associated with a reduced risk of overall cancer and specific cancer sites. Participants with healthy sleep and lifestyle patterns had a lower risk of overall cancer (HR = 0.72, 95% CI = 0.68–0.77), liver cancer (HR = 0.53, 95% CI = 0.31–0.90), bladder cancer (HR = 0.61, 95% CI = 0.47–0.79), lung cancer (HR = 0.22, 95% CI = 0.19–0.27), and colorectal cancer (HR = 0.80, 95% CI = 0.66–0.96) compared to those with unhealthy patterns.

**Conclusion:**

Our findings highlight the importance of public health education and interventions to improve sleep and other lifestyle behaviours for cancer prevention.

## Introduction

Sleep disorders are increasingly prevalent worldwide [1] and have been linked to numerous chronic health problems like obesity [2], cardiovascular disease [3] and cancers [4–8]. Sleep quality and duration are two main contributors. Previous studies have revealed that long or short sleep duration [4, 5, 8–10], poor sleep quality such as insomnia [11–13] and snoring [14] potentially increase the risk of cancers. However, sleep is multidimensional, and sleep duration, timing, continuity, and sleepiness may interconnect with each other [15] and the comprehensive impact of sleep factors on the risk of cancer remains to be explored.

Other ordinary lifestyle factors, including smoking, alcohol intake, diet habits, as well as exercise engagement, were also related to cancer risk with substantial evidence [16–18]. Lacking physical activity is a well-recognized contributor to various cancers, including colorectal cancer and breast cancer [19]. These lifestyle factors may also have an impact on sleep health. For example, excessive alcohol consumption could disrupt sleep homeostasis, leading to sleep disruptions like snoring [20, 21]. Cigarette smoking has potential associations with poor sleep quality [22, 23]. Moreover, physical exercise has been identified as one of the effective non-pharmacological sleep health interventions [24, 25]. Diet, especially fruit and vegetable intake, may influence sleep quality through the biogenesis of melatonin or polyphenol content [26, 27]. Therefore, the effect of sleep health and other lifestyle factors on cancer risks should be investigated jointly to better explain their roles in cancer development.

In this study, the individual impact of sleep, as well as other lifestyle factors on overall cancer and 20 types of cancers, were initially examined. Subsequently, the combined influence of sleep and lifestyle patterns on cancer risks was also investigated. By converting sleep and other lifestyle factors into measurable scores, we hope to provide a comprehensible explanation of their joint effects on cancer and support guidance on healthy sleep and lifestyle behaviours for reducing cancer risks.

## Methods

### Participants

The study population was from the UK Biobank, a large prospective cohort study involving over 500,000 British people between the ages of 37 and 73 years. Eligible participants attended assessment centers across the UK during the baseline assessment period from 2006 to 2010, where they filled in questionnaires and underwent physical measurements. Longitudinal follow-up was facilitated by connecting to national medical and mortality records. Details about the study design and methodology have been described [28]. In this study, participants who had prevalent cancer at baseline were not included (n = 28,699). Subsequently, participants lacking data on the primary exposures to five sleep traits (n = 86,508) or four lifestyle factors (n = 7,118) were also excluded, yielding a participant sample of 380,042 for later analysis (Supplementary Figure S1).

### Sleep Traits Assessment, Sleep Score Calculation and Sleep Pattern Definition

According to the touchscreen questionnaires collected at baseline, five sleep traits were measured, which consisted of sleep duration, chronotype, insomnia, daytime dozing, and snoring. The detailed questionnaire, along with definitions of each item, can be found in Supplementary Table S1. In brief, we defined a sleep duration of 7–8 h each day as a healthy sleep factor, while sleep durations longer or shorter were considered unhealthy. The preference for a morning chronotype was categorized as a healthy sleep factor. Additionally, participants who reported not having usual insomnia symptoms were defined as exhibiting a healthy sleep factor, and participants who did not receive complaints about snoring from others were also classified as having a healthy sleep factor. Moreover, the absence of excessive daytime dozing was considered a healthy sleep factor. Participants were assigned a score of 1 for each sleep factor when their answer was categorized as “healthy sleep factor” and otherwise a score of 0. A healthy sleep score was generated based on the summation of five individual scores of healthy sleep factor, which ranged between 0 and 5. A healthy sleep pattern was characterized by a total sleep score of 4 or higher, whereas an unhealthy sleep pattern was characterized by a score of 3 or lower [15].

### Lifestyle Factor Assessment, Lifestyle Score Calculation and Lifestyle Pattern Definition

This study evaluated four lifestyle factors by self-reported questionnaire, including cigarette smoking, alcohol consumption, fruit and vegetable intake, and physical activity. These specific factors were selected according to the guidelines established by the World Cancer Research Fund/American Institute of Cancer Research [WCRF/AICR (Arlington, VA)] [29] and had been incorporated into a score in several healthy lifestyle-related studies [29–32]. The definition of healthy lifestyle factors included no current smoking, low to moderate alcohol intake (0–14 UK units/week), regular physical activity (≥600 MET-mins/week), and a diet abundant in fruits and vegetables (over 5 servings daily). Detailed definitions of each item can be found in Supplementary Table S2. Participants were assigned a score of 1 when their answer was categorized as “healthy” for each lifestyle factor and otherwise will be assigned a score of 0. The healthy lifestyle score was determined by totaling the scores of four distinct healthy lifestyle factors, which ranged between 0 and 4. A healthy lifestyle pattern was characterized by a total score of ≥ 3, an intermediate lifestyle pattern was characterized by a total score of 2, and an unhealthy lifestyle pattern was characterized by a total score of ≤1 [15].

### Outcomes

Cancer diagnoses among participants were ascertained through the hospital, national cancer registries, and death records, with cancer cases classified according to the International Classification of Diseases, 9th or 10th revision (ICD9 or ICD10). Participants were follow-up until the endpoint, which was defined as the earliest occurrence of four scenarios: cancer diagnosis, death, loss or the end of the follow-up period (October 2015 in Scotland and March 2016 in England and Wales). Finally, we defined “overall cancer” as all malignant neoplasms except those coding as C44, defined as “Other and unspecified malignant neoplasms of skin.” We also included 20 types of common tumors, encompassing cancers affecting various regions of the body such as the head and neck, esophagus, stomach, colon and rectum, liver, pancreas, breast, uterus, ovary, prostate, lung, kidney, bladder, brain and central nervous system, thyroid, as well as non-Hodgkin’s lymphoma, multiple myeloma, leukemia, melanoma, mesothelioma. The ICD9 and ICD10 codes for each cancer and the numbers of cases are shown in Supplementary Table S3. Participants diagnosed with multiple cancers on the same date (n = 67) were excluded from the analysis of specific types of cancers but were retained as a case for the overall cancer study.

### Statistical Analysis

Baseline characteristics of the study population were described using mean and standard deviation (SD) for continuous variables, along with frequency and the corresponding percentages for categorical data. The follow-up period was calculated from the recruitment date to the earliest date among the four scenarios: cancer diagnosis, death, loss or the end of the follow-up period. The Cox proportional hazard model was used to explore the separate and joint association of sleep and lifestyle patterns with cancer risk. Assumptions of proportional hazards were examined by Schoenfeld residuals. Three models were employed to analyze the link between sleep patterns and cancer risks. As for the first model, only sex and age were used as covariates. The second model was adjusted for sex, age, socioeconomic conditions (measured by Townsend deprivation index, TDI), ethnicity (white, non-white or unknown/missing), sedentary time (sum of hours spent on activities such as television and computer usage, driving per day), employment status (working, retired, or others), education (college, less than college), and body mass index (BMI) (<25, 25–29.9, or ≥30). The third model includes additional covariates of lifestyle pattern (healthy, intermediate, unhealthy). For the analysis of an individual sleep factor, all the other sleep factors were included as covariates. For the analysis of a healthy sleep pattern, the above covariates were used in the second model in addition to sleep pattern (healthy or unhealthy). The Kaplan-Meier plot was employed to illustrate the cumulative cancer incidences across different sleep pattern groups or different lifestyle pattern groups. In the sensitivity analysis, incident cancers that occurred in the first 2 years during follow-up were excluded. The examination of the relationship between sleep pattern and lifestyle pattern with cancer risks was stratified by gender. To mitigate the risk of inflated false-positive results, we utilized the Benjamini-Hochberg procedure and presented adjusted *P* values for the primary analyses. R software (version 4.1.2) was used for all statistical analyses, and *P* < 0.05 was considered statistically significant.

## Results

The number of cases for overall cancer, 20 types of cancers, and controls were grouped by the sleep and lifestyle score and are presented in Supplementary Tables S4, S5. The final study sample included 380,042 participants (24,618 incident cancer cases, mean age = 56.2 years old, 45.6% males). The average follow-up duration was 6.9 years. An overview of the demographical features of the study population is shown in Table 1. Up to 58.4% of participants had a healthy sleep pattern, with this group characterized by a higher proportion of females, younger age, and lower BMI compared to individuals in the unhealthy sleep pattern group. As for socioeconomic factors, the healthy sleep pattern group exhibited lower socioeconomic deprivation, a higher percentage of college degree attainment, and a higher percentage of employment at work. In terms of lifestyle factors, the participants with a healthy sleep pattern had less sedentary time, consume more vegetables and fruits, were less likely to smoke, drink less alcohol, and participate in more physical activities.

**TABLE 1:** Baseline characteristics of the participants (n = 380,042) by sleep patterns (United Kingdom, 2006–2016).

Characteristics	All	Sleep patterns		*P* value
		Healthy (n = 221,913)	Unhealthy (n = 158,129)	
Age, year (mean, SD)	56.23 (8.11)	56.07 (8.22)	56.46 (7.93)	<0.001
Male (n, %)	173,368 (45.62)	95,967 (43.25)	77,401 (48.95)	<0.001
Townsend Deprivation Index (mean, SD)	−1.42 (3.02)	−1.57 (2.94)	−1.23 (3.12)	<0.001
Sedentary time, hour/day (mean, SD)	4.81 (2.39)	4.59 (2.28)	5.11 (2.51)	<0.001
Ethnicity (n, %)				<0.001
White	359,944 (94.71)	210,703 (94.95)	149,241 (94.38)	
Non-white	15,977 (4.20)	8,964 (4.04)	7,013 (4.43)	
Unknown/Missing	4,121 (1.08)	2,246 (1.01)	1,875 (1.19)	
Education (n, %)				<0.001
College	126,594 (33.31)	79,103 (35.65)	47,491 (30.03)	
Less than college	250,628 (65.95)	141,195 (63.63)	109,433 (69.20)	
Missing	2,820 (0.74)	1,615 (0.73)	1,205 (0.76)	
Employment (n, %)				<0.001
Working	225,628 (59.37)	134,969 (60.82)	90,659 (57.33)	
Retired	121,561 (31.99)	70,456 (31.75)	51,105 (32.32)	
Other	32,853 (8.64)	16,488 (7.43)	16,365 (10.35)	
BMI, kg/m^2^ (n, %)				<0.001
<25	126,215 (33.21)	83,693 (37.71)	42,522 (26.89)	
25–29.9	161,448 (42.48)	93,748 (42.25)	67,700 (42.81)	
≥30	90,592 (23.84)	43,532 (19.62)	47,060 (29.76)	
Missing	1,787 (0.47)	940 (0.42)	847 (0.54)	
Smoking (n, %)				<0.001
Never	208,255 (54.80)	129,582 (58.39)	78,673 (49.75)	
Former	132,349 (34.82)	73,667 (33.20)	58,682 (37.11)	
Current	39,438 (10.38)	18,664 (8.41)	20,774 (13.14)	
Drink alcohol, UK unit (n, %)				<0.001
≤14	204,408 (53.79)	122,256 (55.09)	82,152 (51.95)	
14–28	91,764 (24.15)	55,401 (24.97)	36,363 (23.00)	
≥28	83,870 (22.07)	44,256 (19.94)	39,614 (25.05)	
Fruit and vegetable intake serving (n, %)				<0.001
≥5	158,786 (41.78)	96,069 (43.29)	62,717 (39.66)	
2–5	193,195 (50.84)	112,435 (50.67)	80,760 (51.07)	
<2	28,061 (7.38)	13,409 (6.04)	14,652 (9.27)	
Physical activity, MET-mins/week (n, %)				<0.001
≥1,800	132,645 (34.90)	80,813 (36.42)	51,832 (32.78)	
600–1,800	134,895 (35.49)	81,128 (36.56)	53,767 (34.00)	
0–600	112,502 (29.60)	59,972 (27.03)	52,530 (33.22)	
Healthy lifestyle pattern				<0.001
Healthy	245,911 (64.71)	151,829 (68.42)	94,082 (59.50)	
Intermediate	103,832 (27.32)	56,362 (25.40)	47,470 (30.02)	
Unhealthy	30,299 (7.97)	13,722 (6.18)	16,577 (10.48)	

SD, standard deviation; BMI, body mass index; MET, metabolic equivalent.

### The Relationship Between Healthy Sleep, Lifestyle Factors and Cancers

The associations between sleep pattern and lifestyle pattern with the risk of cancer are displayed in Table 2 and Supplementary Tables S6, S7. The overall cancer risk was associated with both healthy sleep pattern (HR = 0.95, 95% CI = 0.93–0.98) and healthy lifestyle pattern (HR = 0.77, 95% CI = 0.73–0.80). Additionally, the healthy sleep and lifestyle pattern also showed protective effects on four types of cancer, including liver cancer (HR = 0.73, 95% CI = 0.57–0.94 for healthy sleep pattern; HR = 0.65, 95% CI = 0.44–0.95 for healthy lifestyle pattern), bladder cancer (HR = 0.82, 95% CI = 0.73–0.93 for healthy sleep pattern; HR = 0.70, 95% CI = 0.58–0.86 for healthy lifestyle pattern), lung cancer (HR = 0.90, 95% CI = 0.81–0.99 for healthy sleep pattern; HR = 0.24, 95% CI = 0.21–0.28 for healthy lifestyle pattern) and colorectal cancer (HR = 0.92, 95% CI = 0.85–0.99 for healthy sleep pattern; HR = 0.86, 95% CI = 0.74–0.98 for healthy lifestyle pattern). The cumulative incidences of cancers in different sleep pattern and lifestyle pattern subgroups are detailed in Supplementary Figures S2, S3.

**TABLE 2:** Associations of high-risk groups of sleep pattern, sleep factors, lifestyle pattern, and lifestyle factors with overall cancer, liver cancer, bladder cancer, lung cancer and colorectal cancer (United Kingdom, 2006–2016).

Exposure	%^a^	Overall cancer		Liver cancer		Bladder cancer		Lung cancer		Colorectal cancer	
		HR (95% CI)^b^	*P* value	HR (95% CI)^b^	*P* value	HR (95% CI)^b^	*P* value	HR (95% CI)^b^	*P* value	HR (95% CI)^b^	*P* value
Sleep patterns											
Unhealthy sleep pattern	41.61	Reference	—	Reference	—	Reference	—	Reference	—	Reference	—
Healthy sleep pattern	58.39	**0.95 (0.93, 0.98)**	**<0.001**	**0.73 (0.57, 0.94)**	**0.015**	**0.82 (0.73, 0.93)**	**0.002**	**0.90 (0.81, 0.99)**	**0.030**	**0.92 (0.85, 0.99)**	**0.036**
Healthy sleep factors											
Healthy sleep duration	68.46	0.99 (0.97, 1.02)	0.664	**0.73 (0.57, 0.95)**	**0.020**	**0.87 (0.76, 0.99)**	**0.032**	**0.89 (0.80, 0.99)**	**0.030**	0.93 (0.85, 1.01)	0.066
Morning chronotype	62.74	**0.96 (0.94, 0.99)**	**0.003**	0.98 (0.75, 1.27)	0.863	0.94 (0.83, 1.07)	0.368	**0.87 (0.79, 0.97)**	**0.009**	0.98 (0.90, 1.06)	0.579
No usual insomnia	72.61	**0.97 (0.94, 1.00)**	**0.045**	0.78 (0.60, 1.02)	0.067	0.93 (0.81, 1.07)	0.297	0.98 (0.88, 1.09)	0.659	1.09 (1.00, 1.19)	0.060
No self-reported snoring	62.70	0.99 (0.96, 1.01)	0.322	1.08 (0.83, 1.40)	0.555	0.90 (0.79, 1.02)	0.089	0.98 (0.89, 1.09)	0.769	0.96 (0.88, 1.04)	0.293
No excessive daytime dozing	97.31	0.93 (0.87, 1.00)	0.051	0.67 (0.40, 1.15)	0.145	1.03 (0.73, 1.44)	0.870	0.92 (0.71, 1.19)	0.536	0.91 (0.74, 1.13)	0.385
Lifestyle patterns											
Unhealthy	7.97	Reference	—	Reference	—	Reference	—	Reference	—	Reference	—
Intermediate	27.32	**0.84 (0.80, 0.88)**	**<0.001**	**0.65 (0.43, 0.98)**	**0.041**	**0.78 (0.63, 0.96)**	**0.019**	**0.44 (0.38, 0.50)**	**<0.001**	1.00 (0.87, 1.16)	0.968
Healthy	64.71	**0.77 (0.73, 0.80)**	**<0.001**	**0.65 (0.44, 0.95)**	**0.028**	**0.70 (0.58, 0.86)**	**<0.001**	**0.24 (0.21, 0.28)**	**<0.001**	**0.86 (0.74, 0.98)**	**0.028**
Healthy lifestyle factors											
Not current smoker	89.62	**0.73 (0.70, 0.76)**	**<0.001**	0.82 (0.56, 1.19)	0.295	**0.53 (0.45, 0.63)**	**<0.001**	**0.18 (0.17, 0.20)**	**<0.001**	0.92 (0.81, 1.05)	0.218
Low/moderate alcohol consumption	77.93	**0.95 (0.92, 0.98)**	**<0.001**	1.09 (0.81, 1.47)	0.573	1.07 (0.93, 1.23)	0.356	**0.85 (0.76, 0.95)**	**0.004**	**0.76 (0.69, 0.82)**	**<0.001**
Regular physical activity	70.40	**0.97 (0.94, 0.99)**	**0.015**	**0.65 (0.50, 0.84)**	**<0.001**	1.04 (0.91, 1.20)	0.557	**0.85 (0.77, 0.95)**	**0.003**	1.00 (0.91, 1.08)	0.925
Healthy diet	41.78	**0.96 (0.94, 0.99)**	**0.002**	1.06 (0.82, 1.37)	0.654	0.93 (0.82, 1.06)	0.296	**0.86 (0.77, 0.95)**	**0.004**	1.01 (0.94, 1.10)	0.732

^a^ Percentage referred to the proportion of healthy responders for a given sleep factor among 380,042 participants.

^b^ The hazard ratios (HRs), corresponding 95% confidence intervals (CIs) and *P* values were calculated using Cox regression model adjusting for sex, age, Townsend Deprivation Index, ethnicity, sedentary time, employment, education, BMI, and mutually adjusted for sleep pattern or lifestyle pattern as appropriate.

Bold values provided *P* value < 0.05.

The sensitivity study involved excluding incident cancer cases within the first 2 years following the baseline. Consistent and significant effects on overall cancer risk were observed with adherence to a healthy sleep or lifestyle pattern (HR = 0.95, 95% CI = 0.92–0.98 for healthy sleep pattern; HR = 0.77, 95% CI = 0.73–0.81 for healthy lifestyle pattern). Similar consistent results were also observed for bladder and lung cancers. While the correlation between liver cancer and the healthy lifestyle pattern remained significant, the association between liver cancer and the healthy sleep pattern was no longer significant. In addition, the relationship between colorectal cancer and sleep pattern remained statistically significant, whereas no significant association between colorectal cancer and lifestyle pattern was found (Supplementary Tables S8, S9).

Stratification analysis by gender was conducted and the results are shown in Supplementary Tables S10, S11. The association of healthy sleep pattern and healthy lifestyle pattern with overall cancer remained statistically significant in both males and females. For females, the associations of healthy sleep and lifestyle patterns with lung cancer risk were consistently observed, while no statistical significance was observed for liver, colorectal and bladder cancer. For males, healthy sleep pattern was significantly associated with colorectal cancer and bladder cancer, and healthy lifestyle pattern was significantly associated with liver, bladder and lung cancers.

Using the healthy sleep score and as healthy lifestyle score continuous variables, consistent results were found on overall cancer, as well as bladder, liver and lung cancer in Model 3. However, no association between healthy sleep and lifestyle factors with colorectal cancer risk was found (Supplementary Tables S12, S13). After excluding incident cancer cases in the first 2 years, the associations remained statistically significant except for liver cancer. However, no significant association was observed between the two scores and colorectal cancer, and this result should be interpreted with caution.

We also evaluated the effects of various healthy sleep factors and healthy lifestyle factors on the risk of overall cancer and the likelihood of developing four cancers, including liver cancer, bladder cancer, lung cancer and colorectal cancer (Table 2). We found that individuals with morning chronotype (HR = 0.96, 95% CI = 0.94–0.99) or no usual insomnia (HR = 0.97, 95% CI = 0.94–1.00) showed a reduced risk of overall cancer. Besides, a healthy sleep duration was linked to a reduced liver cancer risk (HR = 0.73, 95% CI = 0.57–0.95), bladder cancer risk (HR = 0.87, 95% CI = 0.76–0.99), and lung cancer risk (HR = 0.89, 95% CI = 0.80–0.99). Furthermore, all four lifestyle factors showed significant protective effects on both overall cancer and lung cancer. Results for the overall cancer, as well as all 20 specific cancer sites, are displayed in Supplementary Figures S4, S5.

### Joint Association of Sleep Pattern and Lifestyle Pattern With Cancers

The joint association of sleep pattern and lifestyle pattern with cancers was additionally evaluated. Compared with the unhealthy sleep - unhealthy lifestyle group, participants with healthy sleep and healthy lifestyle pattern could reduce the risk of overall cancer by 28% (HR = 0.72, 95% CI = 0.68–0.77), liver cancer by 47% (HR = 0.53, 95% CI = 0.31–0.90), bladder cancer by 39% (HR = 0.61,0.47–0.79), lung cancer by 78% (HR = 0.22, 95% CI = 0.19–0.27), and colorectal cancer by 20% (HR = 0.80, 95% CI = 0.66–0.96) (Table 3). After excluding cancer cases diagnosed in the first 2 years post-baseline, consistent results were observed (Supplementary Table S14).

**TABLE 3:** The joint associations of sleep pattern and healthy lifestyle pattern with overall cancer, liver cancer, bladder cancer, lung cancer and colorectal cancer (United Kingdom, 2006–2016).

Sleep pattern	Lifestyle pattern	Overall cancer		Liver cancer		Bladder cancer		Lung cancer		Colorectal cancer	
		HR (95% CI)^a^	*P* value^a^	HR (95% CI)^a^	*P* value^a^	HR (95% CI)^a^	*P* value^a^	HR (95% CI)^a^	*P* value^a^	HR (95% CI)^a^	*P* value^a^
Unhealthy	Unhealthy	—	—	—	—	—	—	—	—	—	—
	Intermediate	**0.82 (0.77, 0.87)**	**<0.001**	0.71 (0.40, 1.28)	0.260	0.83 (0.63, 1.11)	0.208	**0.47 (0.39, 0.56)**	**<0.001**	1.02 (0.84, 1.24)	0.835
	Healthy	**0.77 (0.72, 0.82)**	**<0.001**	0.91 (0.54, 1.55)	0.732	**0.75 (0.57, 0.97)**	**0.032**	**0.24 (0.20, 0.29)**	**<0.001**	0.86 (0.71, 1.04)	0.125
Healthy	Unhealthy	0.95 (0.87, 1.03)	0.181	1.26 (0.64, 2.50)	0.505	0.94 (0.66, 1.35)	0.734	0.96 (0.78, 1.18)	0.686	0.94 (0.73, 1.22)	0.662
	Intermediate	**0.81 (0.76, 0.86)**	**<0.001**	0.69 (0.38, 1.24)	0.212	**0.66 (0.49, 0.88)**	**0.005**	**0.39 (0.32, 0.47)**	**<0.001**	0.93 (0.76, 1.13)	0.454
	Healthy	**0.72 (0.68, 0.77)**	**<0.001**	**0.53 (0.31, 0.90)**	**0.020**	**0.61 (0.47, 0.79)**	**<0.001**	**0.22 (0.19, 0.27)**	**<0.001**	**0.80 (0.66, 0.96)**	**0.017**

^a^ The hazard ratios (HRs), corresponding 95% confidence intervals (CIs) and *P* values were calculated using the Cox regression model adjusting for sex, age, Townsend Deprivation Index, ethnicity, sedentary time, employment, education, and BMI.

Bold values provided *P* value < 0.05.

## Discussion

This study provides new insights into the relationship between sleep patterns, lifestyle patterns, and cancer risk. Using the UK Biobank, one of the largest cohort studies, we examined the individual effects of sleep and lifestyle patterns on the risk of overall cancer and 20 cancer types, as well as the combined impact of these patterns on cancer development. By constructing separate comprehensive scores for sleep and lifestyle patterns, we found that participants with healthier sleep or lifestyle patterns had reduced risks of overall cancer and four specific types—liver, bladder, lung, and colorectal cancers. The joint association analysis consistently supported these findings.

The interplay of sleep timing, quality and duration is widely recognized. They jointly interpret healthy sleep patterns and contribute to many diseases. For example, insomnia and late chronotype might lead to oversleepiness during the day, insufficient sleep duration, and disturbed circadian rhythm [15, 33]. Sleep duration and severe insomnia have joint effects on cardiovascular episodes and overall mortality [34]. Therefore, we suggest that building a comprehensive sleep score should better reflect the overall sleep patterns and interpret the relationship with cancer outcomes. The findings of our research indicated that having a healthy sleep behaviour could decrease the risk of overall cancer, along with colorectal, bladder, lung and liver cancer. In particular, sleep duration, quality and timing (chronotype and insomnia) all contribute to cancer risk, suggesting that promoting healthy sleep for cancer prevention should consider improving sleep duration and quality. Nevertheless, some associations did not retain statistical significance in the sensitivity or stratification analysis and warrant careful explanation, e.g., no association between sleep patterns and liver cancer after excluding incident cancers diagnosed within the first 2 years of follow-up, no association between sleep patterns and liver cancer when stratified by gender. In addition, some unexpected results were observed in our study and should be explained with caution. For example, a previous study used sleep duration as a continuous variable and found prolonged sleep duration was associated with increased breast cancer risk [35]. In our study, we used a different categorization method by comparing healthy sleep duration (7–8 h per day) with shorter or longer sleep durations and found a lower risk for the shorter or longer sleep time group. This discrepant observation may be explained by the “J-shape” relation between sleep duration and breast cancer risk [36]. We also performed an analysis treating sleep duration as a continuous variable and found a risk effect of prolonged sleep time (HR = 1.03, 95% CI = 1.00–1.06 per hour increase, data not shown), which was consistent with the previous studies. Besides, unexpected results were also observed in some sleep factors and cancer risk, e.g., increased corpus uteri and ovarian cancer risk for no usual insomnia and increased ovarian cancer risk for no self-reported snoring. Individuals with usual insomnia may use medications or related therapies (e.g., melatonin supplements), which could have protective effects against cancer through hormonal modulation [37–39]. Snoring is often a symptom of obstructive sleep apnea (OSA), which can disrupt hormonal cycles and lower estrogen exposure levels, which may reduce the risk of hormone-related cancers like ovarian cancer [40, 41]. Since the numbers of corpus uteri and ovarian cancer are small in this study (N = 684 and 471), further study is warranted to investigate the relationship between the two sleep factors and hormone-related cancers risk.

Moreover, studies have suggested certain unhealthy lifestyle factors, such as low vegetable intake [26], alcohol consumption [42], and smoking [43], could exacerbate the negative effects of sleep behaviours on health risks, while physical activity has been identified as an essential strategy to improve sleep health [24, 25]. The synergistic effects of sleep behaviours and other lifestyle behaviour are worth investigated. In this study, we found maintaining healthy sleep and lifestyle patterns could jointly decrease the risk of overall cancer and four types of cancers: colorectal, bladder, lung, and liver cancer. This finding highlights the importance of interventions targeting both sleep (e.g., early bedtimes, appropriate sleep duration) and other modifiable lifestyle behaviours (e.g., healthy diet, smoking cessation) for cancer prevention. Nevertheless, some unexpected results were found between lifestyle factors and cancer risks, e.g., increased corpus uteri cancer and melanoma risk for no current smoking, increased kidney cancer and non-Hodgkin lymphoma risk for low/moderate alcohol consumption. These unexpected results were also observed in the previous study. For example, the association of smoking with lower corpus uteri cancer risk was supported by several studies [44, 45]. This observation may be explained by the effect of nicotine on increasing estrogen metabolism and lowering the level of circulating estrogen [46, 47]. The association of smoking with lower melanoma risk was also consistent with a recent review and meta-analysis [48]. The proposed explanation was the reduced cutaneous inflammatory response seen in smokers, which modifies deleterious UV-induced cutaneous immune reactions [49–53]. Alcohol drinking was associated with lower kidney cancer and non-Hodgkin lymphoma risk, which was supported by a systematic review and meta-analysis [54, 55], though the lack of a biological explanation suggests caution in the interpretation of results.

There are several limitations of this study. Firstly, the majority of the study population is White, suggesting more caution when extending the conclusion to other ethnic groups. Secondly, the assessment of sleep traits and other lifestyles was based on a questionnaire and measured at baseline, and a relatively short follow-up time might not have captured the longtime influence of sleep and lifestyle habits on cancer risk. Dynamic evaluation using recorded data from the device is warranted in future studies when available data is obtained. Lastly, the healthy diet factor of this study was only considered based on nutrient intake, but for future research, adhering to nutritious eating habits, such as the Mediterranean diet or following dietary recommendations, is also valuable.

In summary, healthy sleep behaviour and healthy lifestyle jointly contribute to a reduced risk of overall and site-specific cancers, including liver, bladder, lung and colorectal cancer. Health education for keeping a healthy lifestyle or intervention for poor sleep behaviour would be valuable for cancer prevention.

## References

[1] Stranges S Tigbe W Gómez-Olivé FX Thorogood M Kandala NB. Sleep Problems: An Emerging Global Epidemic? Findings From the INDEPTH WHO-SAGE Study Among More Than 40,000 Older Adults From 8 Countries Across Africa and Asia. Sleep (2012) 35:1173–81. 10.5665/sleep.2012 22851813 PMC3397790

[2] Cappuccio FP Taggart FM Kandala NB Currie A Peile E Stranges S Meta-Analysis of Short Sleep Duration and Obesity in Children and Adults. Sleep (2008) 31:619–26. 10.1093/sleep/31.5.619 18517032 PMC2398753

[3] Cappuccio FP Cooper D D’Elia L Strazzullo P Miller MA. Sleep Duration Predicts Cardiovascular Outcomes: A Systematic Review and Meta-Analysis of Prospective Studies. Eur Heart J (2011) 32:1484–92. 10.1093/eurheartj/ehr007 21300732

[4] Song C Zhang R Wang C Fu R Song W Dou K Sleep Quality and Risk of Cancer: Findings From the English Longitudinal Study of Aging. Sleep (2021) 44:zsaa192. 10.1093/sleep/zsaa192 32954418 PMC7953221

[5] Chen Y Tan F Wei L Li X Lyu Z Feng X Sleep Duration and the Risk of Cancer: A Systematic Review and Meta-Analysis Including Dose-Response Relationship. BMC Cancer (2018) 18:1149. 10.1186/s12885-018-5025-y 30463535 PMC6249821

[6] Manouchehri E Taghipour A Ghavami V Ebadi A Homaei F Latifnejad Roudsari R. Night-Shift Work Duration and Breast Cancer Risk: An Updated Systematic Review and Meta-Analysis. BMC Womens Health (2021) 21:89. 10.1186/s12905-021-01233-4 33653334 PMC7927396

[7] Mogavero MP DelRosso LM Fanfulla F Bruni O Ferri R. Sleep Disorders and Cancer: State of the Art and Future Perspectives. Sleep Med Rev (2021) 56:101409. 10.1016/j.smrv.2020.101409 33333427

[8] Titova OE Michaëlsson K Vithayathil M Mason AM Kar S Burgess S Sleep Duration and Risk of Overall and 22 Site-Specific Cancers: A Mendelian Randomization Study. Int J Cancer (2021) 148:914–20. 10.1002/ijc.33286 32895918 PMC7821333

[9] Cao J Eshak ES Liu K Muraki I Cui R Iso H Sleep Duration and Risk of Breast Cancer: The JACC Study. Breast Cancer Res Treat (2019) 174:219–25. 10.1007/s10549-018-4995-4 30460465

[10] Jiao L Duan Z Sangi-Haghpeykar H Hale L White DL El-Serag HB. Sleep Duration and Incidence of Colorectal Cancer in Postmenopausal Women. Br J Cancer (2013) 108:213–21. 10.1038/bjc.2012.561 23287986 PMC3553538

[11] Fang HF Miao NF Chen CD Sithole T Chung MH. Risk of Cancer in Patients With Insomnia, Parasomnia, and Obstructive Sleep Apnea: A Nationwide Nested Case-Control Study. J Cancer (2015) 6:1140–7. 10.7150/jca.12490 26516362 PMC4615350

[12] Sen A Opdahl S Strand LB Vatten LJ Laugsand LE Janszky I. Insomnia and the Risk of Breast Cancer: The HUNT Study. Psychosom Med (2017) 79:461–8. 10.1097/psy.0000000000000417 27763987

[13] Shi T Min M Sun C Zhang Y Liang M Sun Y. Does Insomnia Predict a High Risk of Cancer? A Systematic Review and Meta-Analysis of Cohort Studies. J Sleep Res (2020) 29:e12876. 10.1111/jsr.12876 31352687

[14] Zhang X Giovannucci EL Wu K Gao X Hu F Ogino S Associations of Self-Reported Sleep Duration and Snoring With Colorectal Cancer Risk in Men and Women. Sleep (2013) 36:681–8. 10.5665/sleep.2626 23633750 PMC3624822

[15] Fan M Sun D Zhou T Heianza Y Lv J Li L Sleep Patterns, Genetic Susceptibility, and Incident Cardiovascular Disease: A Prospective Study of 385 292 UK Biobank Participants. Eur Heart J (2020) 41:1182–9. 10.1093/eurheartj/ehz849 31848595 PMC7071844

[16] Katzke VA Kaaks R Kühn T. Lifestyle and Cancer Risk. Cancer J (2015) 21:104–10. 10.1097/ppo.0000000000000101 25815850

[17] Islami F Goding Sauer A Miller KD Siegel RL Fedewa SA Jacobs EJ Proportion and Number of Cancer Cases and Deaths Attributable to Potentially Modifiable Risk Factors in the United States. CA Cancer J Clin (2018) 68:31–54. 10.3322/caac.21440 29160902

[18] Zhu Y Yang H Liang S Zhang H Mo Y Rao S Higher Adherence to Healthy Lifestyle Score Is Associated With Lower Odds of Non-Alcoholic Fatty Liver Disease. Nutrients (2022) 14:4462. 10.3390/nu14214462 36364725 PMC9657000

[19] Brown JC Winters-Stone K Lee A Schmitz KH. Cancer, Physical Activity, and Exercise. Compr Physiol (2012) 2:2775–809. 10.1002/cphy.c120005 23720265 PMC4122430

[20] Ebrahim IO Shapiro CM Williams AJ Fenwick PB. Alcohol and Sleep I: Effects on Normal Sleep. Alcohol Clin Exp Res (2013) 37:539–49. 10.1111/acer.12006 23347102

[21] Thakkar MM Sharma R Sahota P. Alcohol Disrupts Sleep Homeostasis. Alcohol (2015) 49:299–310. 10.1016/j.alcohol.2014.07.019 25499829 PMC4427543

[22] Zandy M Chang V Rao DP Do MT. Tobacco Smoke Exposure and Sleep: Estimating the Association of Urinary Cotinine With Sleep Quality. Health Promot Chronic Dis Prev Can (2020) 40:70–80. 10.24095/hpcdp.40.3.02 32162509 PMC7093068

[23] Truong MK Berger M Haba-Rubio J Siclari F Marques-Vidal P Heinzer R. Impact of Smoking on Sleep Macro- and Microstructure. Sleep Med (2021) 84:86–92. 10.1016/j.sleep.2021.05.024 34126401

[24] Murawski B Wade L Plotnikoff RC Lubans DR Duncan MJ. A Systematic Review and Meta-Analysis of Cognitive and Behavioral Interventions to Improve Sleep Health in Adults Without Sleep Disorders. Sleep Med Rev (2018) 40:160–9. 10.1016/j.smrv.2017.12.003 29397329

[25] Yang PY Ho KH Chen HC Chien MY. Exercise Training Improves Sleep Quality in Middle-Aged and Older Adults With Sleep Problems: A Systematic Review. J Physiother (2012) 58:157–63. 10.1016/s1836-9553(12)70106-6 22884182

[26] Zuraikat FM Wood RA Barragán R St-Onge MP. Sleep and Diet: Mounting Evidence of a Cyclical Relationship. Annu Rev Nutr (2021) 41:309–32. 10.1146/annurev-nutr-120420-021719 34348025 PMC8511346

[27] Noorwali E Hardie L Cade J. Bridging the Reciprocal Gap Between Sleep and Fruit and Vegetable Consumption: A Review of the Evidence, Potential Mechanisms, Implications, and Directions for Future Work. Nutrients (2019) 11:1382. 10.3390/nu11061382 31248175 PMC6627504

[28] Sudlow C Gallacher J Allen N Beral V Burton P Danesh J UK Biobank: An Open Access Resource for Identifying the Causes of a Wide Range of Complex Diseases of Middle and Old Age. Plos Med (2015) 12:e1001779. 10.1371/journal.pmed.1001779 25826379 PMC4380465

[29] Shams-White MM Brockton NT Mitrou P Romaguera D Brown S Bender L Operationalizing the 2018 World Cancer Research Fund/American Institute for Cancer Research (WCRF/AICR) Cancer Prevention Recommendations: A Standardized Scoring System. Nutrients (2019) 11 (7):1572. 10.3390/nu11071572 31336836 PMC6682977

[30] Zhu M Wang T Huang Y Zhao X Ding Y Zhu M Genetic Risk for Overall Cancer and the Benefit of Adherence to a Healthy Lifestyle. Cancer Res (2021) 81:4618–27. 10.1158/0008-5472.Can-21-0836 34321244

[31] Chudasama YV Khunti K Gillies CL Dhalwani NN Davies MJ Yates T Healthy Lifestyle and Life Expectancy in People With Multimorbidity in the UK Biobank: A Longitudinal Cohort Study. Plos Med (2020) 17:e1003332. 10.1371/journal.pmed.1003332 32960883 PMC7508366

[32] Lv J Yu C Guo Y Bian Z Yang L Chen Y Adherence to Healthy Lifestyle and Cardiovascular Diseases in the Chinese Population. J Am Coll Cardiol (2017) 69:1116–25. 10.1016/j.jacc.2016.11.076 28254173 PMC6675601

[33] Zhou T Yuan Y Xue Q Li X Wang M Ma H Adherence to a Healthy Sleep Pattern Is Associated With Lower Risks of All-Cause, Cardiovascular and Cancer-Specific Mortality. J Intern Med (2022) 291:64–71. 10.1111/joim.13367 34237797 PMC8688171

[34] Chien KL Chen PC Hsu HC Su TC Sung FC Chen MF Habitual Sleep Duration and Insomnia and the Risk of Cardiovascular Events and All-Cause Death: Report From a Community-Based Cohort. Sleep (2010) 33:177–84. 10.1093/sleep/33.2.177 20175401 PMC2817905

[35] Richmond RC Anderson EL Dashti HS Jones SE Lane JM Strand LB Investigating Causal Relations Between Sleep Traits and Risk of Breast Cancer in Women: Mendelian Randomisation Study. Bmj (2019) 365:l2327. 10.1136/bmj.l2327 31243001 PMC6592406

[36] Lu C Sun H Huang J Yin S Hou W Zhang J Long-Term Sleep Duration as a Risk Factor for Breast Cancer: Evidence From a Systematic Review and Dose-Response Meta-Analysis. Biomed Res Int (2017) 2017:4845059. 10.1155/2017/4845059 29130041 PMC5654282

[37] Li Y Li S Zhou Y Meng X Zhang JJ Xu DP Melatonin for the Prevention and Treatment of Cancer. Oncotarget (2017) 8:39896–921. 10.18632/oncotarget.16379 28415828 PMC5503661

[38] Xie Z Chen F Li WA Geng X Li C Meng X A Review of Sleep Disorders and Melatonin. Neurol Res (2017) 39:559–65. 10.1080/01616412.2017.1315864 28460563

[39] Viswanathan AN Schernhammer ES. Circulating Melatonin and the Risk of Breast and Endometrial Cancer in Women. Cancer Lett (2009) 281:1–7. 10.1016/j.canlet.2008.11.002 19070424 PMC2735793

[40] Netzer NC Eliasson AH Strohl KP. Women With Sleep Apnea Have Lower Levels of Sex Hormones. Sleep Breath (2003) 7:25–9. 10.1007/s11325-003-0025-8 12712394

[41] Sigurðardóttir ES Gislason T Benediktsdottir B Hustad S Dadvand P Demoly P Female Sex Hormones and Symptoms of Obstructive Sleep Apnea in European Women of a Population-Based Cohort. PLoS One (2022) 17:e0269569. 10.1371/journal.pone.0269569 35731786 PMC9216532

[42] Kurhaluk N. Alcohol and Melatonin. Chronobiol Int (2021) 38:785–800. 10.1080/07420528.2021.1899198 33761823

[43] Zeng X Ren Y Wu K Yang Q Zhang S Wang D Association Between Smoking Behavior and Obstructive Sleep Apnea: A Systematic Review and Meta-Analysis. Nicotine Tob Res (2023) 25:364–71. 10.1093/ntr/ntac126 35922388 PMC9910143

[44] Zhou B Yang L Sun Q Cong R Gu H Tang N Cigarette Smoking and the Risk of Endometrial Cancer: A Meta-Analysis. Am J Med (2008) 121:501–8. 10.1016/j.amjmed.2008.01.044 18501231

[45] Felix AS Yang HP Gierach GL Park Y Brinton LA. Cigarette Smoking and Endometrial Carcinoma Risk: The Role of Effect Modification and Tumor Heterogeneity. Cancer Causes Control (2014) 25:479–89. 10.1007/s10552-014-0350-1 24487725 PMC4151521

[46] Geisler J Omsjø IH Helle SI Ekse D Silsand T Lønning PE. Plasma Oestrogen Fractions in Postmenopausal Women Receiving Hormone Replacement Therapy: Influence of Route of Administration and Cigarette Smoking. J Endocrinol (1999) 162:265–70. 10.1677/joe.0.1620265 10425465

[47] Jandíková H Dušková M Stárka L. The Influence of Smoking and Cessation on the Human Reproductive Hormonal Balance. Physiol Res (2017) 66:S323-S331–s331. 10.33549/physiolres.933724 28948816

[48] Friedman EB Williams GJ Lo SN Thompson JF. Effect of Smoking on Melanoma Incidence: A Systematic Review With Meta-Analysis. J Natl Cancer Inst (2024) 116:1739–52. 10.1093/jnci/djae142 38913874

[49] Odenbro A Gillgren P Bellocco R Boffetta P Håkansson N Adami J. The Risk for Cutaneous Malignant Melanoma, Melanoma *In Situ* and Intraocular Malignant Melanoma in Relation to Tobacco Use and Body Mass Index. Br J Dermatol (2007) 156:99–105. 10.1111/j.1365-2133.2006.07537.x 17199574

[50] Denkert C Köbel M Berger S Siegert A Leclere A Trefzer U Expression of Cyclooxygenase 2 in Human Malignant Melanoma. Cancer Res (2001) 61:303–8. 11196178

[51] Mills CM Hill SA Marks R. Altered Inflammatory Responses in Smokers. Bmj (1993) 307:911. 10.1136/bmj.307.6909.911 8241856 PMC1679069

[52] Hardie CM Elliott F Chan M Rogers Z Bishop DT Newton-Bishop JA. Environmental Exposures Such as Smoking and Low Vitamin D Are Predictive of Poor Outcome in Cutaneous Melanoma Rather Than Other Deprivation Measures. J Invest Dermatol (2020) 140:327–37. 10.1016/j.jid.2019.05.033 31425707 PMC6983339

[53] Mills CM. Cigarette Smoking, Cutaneous Immunity, and Inflammatory Response. Clin Dermatol (1998) 16:589–94. 10.1016/s0738-081x(98)00044-3 9787971

[54] Tramacere I Pelucchi C Bonifazi M Bagnardi V Rota M Bellocco R Alcohol Drinking and Non-Hodgkin Lymphoma Risk: A Systematic Review and a Meta-Analysis. Ann Oncol (2012) 23:2791–8. 10.1093/annonc/mds013 22357444

[55] Song DY Song S Song Y Lee JE. Alcohol Intake and Renal Cell Cancer Risk: A Meta-Analysis. Br J Cancer (2012) 106:1881–90. 10.1038/bjc.2012.136 22516951 PMC3364130

