# Supplementary material for: Sleep Pattern, Lifestyle Pattern, and Risks of Overall and 20 Types of Cancers: Findings From the UK Biobank Cohort
Source: Int J Public Health. 2025 Jan 17;69:1607726. doi: 10.3389/ijph.2024.1607726 (PMC11781944; doi:10.3389/ijph.2024.1607726)
Supplement: Supplementary file 1 [file DataSheet1.docx]

**International Journal of Public Health**

**Sleep pattern, lifestyle pattern, and risks of overall and 20 types of cancers: findings from the UK Biobank cohort**

**Supplementary File**

**List of captions for supplementary tables and figures**

Supplementary Table S1. The assessment of sleep traits and the definition of healthy sleep factors (United Kingdom,2006–2016).

Supplementary Table S2. The assessment of lifestyle and the definition of healthy lifestyle factors (United Kingdom,2006–2016).

Supplementary Table S3. The International Classification of Diseases code of outcomes (United Kingdom,2006–2016).

Supplementary Table S4. The number of participants with different healthy sleep scores (United Kingdom,2006–2016).

Supplementary Table S5. The number of participants with different healthy lifestyle scores (United Kingdom,2006–2016).

Supplementary Table S6. The association of sleep patterns with overall and 20 types of cancers (United Kingdom,2006–2016).

Supplementary Table S7. The association of lifestyle patterns with overall and 20 types of cancers (United Kingdom,2006–2016).

Supplementary Table S8. The association of sleep patterns with overall and 20 types of cancers after excluding cancers occurring within the first 2 years after the baseline (n = 374,038) (United Kingdom,2006–2016).

Supplementary Table S9. The association of intermediate and healthy lifestyle patterns with overall and 20 types of cancers after excluding cancers occurring within the first 2 years after the baseline (n = 374,038) (United Kingdom,2006–2016).

Supplementary Table S10. The association of sleep patterns with overall and 20 types of cancers stratified by sex (United Kingdom,2006–2016).

Supplementary Table S11. The association of lifestyle patterns with overall and 20 types of cancers stratified by sex (United Kingdom,2006–2016).

Supplementary Table S12. The association of the healthy sleep scores as a continuous variable with overall and 20 types of cancers (United Kingdom,2006–2016).

Supplementary Table S13. The association of healthy lifestyle scores as a continuous variable with overall and 20 types of cancers (United Kingdom,2006–2016).

Supplementary Table S14. The joint association of sleep pattern and lifestyle pattern with overall cancer, liver cancer, bladder cancer, lung cancer and colorectal cancer after excluding cancers occurring within the first 2 years after the baseline (N = 374,038) (United Kingdom,2006–2016).

Supplementary Figure S1. Flow chart of participants included in the study (United Kingdom,2006–2016).

Supplementary Figure S2. Cumulative incidences of overall cancer, liver cancer, bladder cancer, lung cancer, and colorectal cancer in the healthy and unhealthy sleep patterns (United Kingdom,2006–2016).

Supplementary Figure S3. Cumulative incidences of overall cancer, liver cancer, bladder cancer, lung cancer, and colorectal cancer in the healthy, intermediate and unhealthy lifestyle patterns (United Kingdom,2006–2016).

Supplementary Figure S4. Associations of sleep factors with overall and 20 types of cancers (United Kingdom,2006–2016).

Supplementary Figure S5. Associations of lifestyle factors with overall and 20 types of cancers (United Kingdom,2006–2016).

**Supplementary Table S1. The assessment of sleep traits and the definition of healthy sleep factors (United Kingdom,2006–2016).**

| **Sleep traits** | **UK Biobank Code** | **UK Biobank Questionnaire** | **Answers classified to a healthy sleep factor (%)** | **Answers classified to an unhealthy sleep factor (%)** |
| --- | --- | --- | --- | --- |
| Sleep duration | 1160 | About how many hours of sleep do you get in every 24 hours? (please include naps) | 7-8 hour/d (68.46%) | ≤6 or ≥9 hour/d (31.54%) |
| Chronotype | 1180 | Do you consider yourself to be? | Definitely a “morning” person; more a “morning” than evening” person (62.74%) | Definitely an “evening” person; more an “evening” than a “morning person” (37.26%) |
| Insomnia | 1200 | Do you have trouble falling asleep at night or do you wake up in the middle of the night? | Never/rarely; sometimes (72.61%) | Usually (27.39%) |
| Snoring | 1210 | Does your partner or a close relative or friend complain about your snoring? | No (62.70%) | Yes (37.30%) |
| Daytime dozing | 1220 | How likely are you to doze off or fall asleep during the daytime when you don't mean to? (e.g. when working, reading, or driving) | Never/rarely; sometimes (97.31%) | Often; all the time (2.69%) |

**Supplementary Table S2. The assessment of lifestyle and the definition of healthy lifestyle factors (United Kingdom,2006–2016).**

| **Lifestyle traits** | **UK Biobank Code** | **Description** | **Answers classified to a healthy lifestyle factor (%)** | **Answers classified to an unhealthy lifestyle factor (%)** |
| --- | --- | --- | --- | --- |
| Cigarette smoking | 20116 | The existing variable ‘smoking status’ (20116) was applied | Never; previous smoker (89.62%) | current smoker (10.38%) |
| Alcohol | 1558, 1568, 1578, 1588, 1598, 1608, 5364, 4407, 4418, 4440, 4451, 4429; 20117 | The level of overall alcohol consumption as the number of UK units of alcohol (10 mL/unit) consumed per week was calculated; participants were categorized based on the consumption according to the UK guideline (14 UK units/week). | 0-14 UK units /week (77.93%) | ≥14 UK units /week (22.07%) |
| Physical activity | 864, 874, 884, 894, 904, 914 | Weekly PA was summarized using the weekly total metabolic equivalent task (MET), calculated by multiplying the MET value of activity by the number of PA hours per week. Participants were categorized based on the amount according to the WHO PA guideline (600 MET-mins/week) | ≥600 MET-mins/week (70.40%) | 0-600 MET-mins/week (29.60%) |
| Diet | 1289, 1299, 1309 | The sum of the daily servings of ’cooked vegetable intake’ (1289), 'salad /raw vegetable intake' (1299), and 'fresh fruit intake' (1309) served as a proxy for a healthy diet. | ≥5 servings (41.78%) | 0-5 servings (58.22%) |

**Supplementary Table S3. The International Classification of Diseases code of outcomes (United Kingdom,2006–2016).**

| **Type of cancer** | **ICD10** | **ICD9** | **Case number** |
| --- | --- | --- | --- |
| **Head and neck** | C00-14, C30-32 | 140-149, 161, 162 | 614 |
| **Esophagus** | C15 | 150 | 416 |
| **Stomach** | C16 | 151 | 290 |
| **Colorectal** | C18-21 | 153-154 | 2718 |
| **Liver** | C22 | 155 | 255 |
| **Pancreas** | C25 | 157 | 488 |
| **Breast** | C50 | 174,175 | 4823 |
| **Corpus uteri** | C54 | 182 | 684 |
| **Ovary** | C56 | 1830 | 471 |
| **Prostate** | C61 | 185 | 4835 |
| **Kidney** | C64-65 | 1890-1891 | 654 |
| **Bladder** | C67 | 188 | 1032 |
| **Non-Hodgkin lymphoma** | C82-86, C96 | 202 | 993 |
| **Multiple myeloma** | C88, C90 | 203 | 385 |
| **Leukemia** | C91-95 | 204-208 | 650 |
| **Lung** | C33-34 | 162 | 1622 |
| **Melanoma** | C43 | 172 | 1419 |
| **Mesothelioma** | C45 | - | 198 |
| **Brain and CNS** | C70-72 | 191,192 | 395 |
| **Thyroid** | C73 | 193 | 206 |
| **Overall cancer** | All malignancies except C44 | 140-172, 174-209 | 24618 |

*ICD, Revision of the International Classification of Diseases; Brain and CNS, brain and central nervous system cancers.*

**Supplementary Table S4. The number of participants with different healthy sleep scores (United Kingdom,2006–2016).**

| **Participants** | **Healthy sleep scores** | | | | |
| --- | --- | --- | --- | --- | --- |
|  | **0-1** | **2** | **3** | **4** | **5** |
| **All participants included in this study** | 9087 (2.39%) | 42434 (11.17%) | 106608 (28.05%) | 140060 (36.85%) | 81853 (21.54%) |
| **Overall cancer** | 650 (2.64%) | 2981 (12.11%) | 7222 (29.34%) | 8863 (36.00%) | 4902 (19.91%) |
| **Head and neck** | 18 (2.93%) | 88 (14.33%) | 177 (28.83%) | 206 (33.55%) | 125 (20.36%) |
| **Esophagus** | 14 (3.37%) | 60 (14.42%) | 132 (31.73%) | 159 (38.22%) | 51 (12.26%) |
| **Stomach** | 11 (3.79%) | 30 (10.34%) | 93 (32.07%) | 110 (37.93%) | 46 (15.86%) |
| **Colorectal** | 62 (2.28%) | 314 (11.55%) | 853 (31.38%) | 952 (35.03%) | 537 (19.76%) |
| **Liver** | 17 (6.67%) | 34 (13.33%) | 86 (33.73%) | 75 (29.41%) | 43 (16.86%) |
| **Pancreas** | 12 (2.46%) | 64 (13.11%) | 135 (27.66%) | 183 (37.50%) | 94 (19.26%) |
| **Breast** | 109 (2.26%) | 518 (10.74%) | 1335 (27.68%) | 1729 (35.85%) | 1132 (23.47%) |
| **Corpus uteri** | 19 (2.78%) | 91 (13.30%) | 190 (27.78%) | 235 (34.36%) | 149 (21.78%) |
| **Ovary** | 4 (0.85%) | 52 (11.04%) | 107 (22.72%) | 195 (41.40%) | 113 (23.99%) |
| **Prostate** | 119 (2.46%) | 567 (11.73%) | 1473 (30.47%) | 1785 (36.92%) | 891 (18.43%) |
| **Kidney** | 16 (2.45%) | 102 (15.60%) | 175 (26.76%) | 237 (36.24%) | 124 (18.96%) |
| **Bladder** | 37 (3.59%) | 149 (14.44%) | 326 (31.59%) | 338 (32.75%) | 182 (17.64%) |
| **Non-Hodgkin lymphoma** | 34 (3.42%) | 118 (11.88%) | 274 (27.59%) | 386 (38.87%) | 181 (18.23%) |
| **Multiple myeloma** | 10 (2.60%) | 43 (11.17%) | 132 (34.29%) | 142 (36.88%) | 58 (15.06%) |
| **Leukemia** | 12 (1.85%) | 81 (12.46%) | 202 (31.08%) | 234 (36.00%) | 121 (18.62%) |
| **Lung** | 67 (4.13%) | 249 (15.35%) | 482 (29.72%) | 543 (33.48%) | 281 (17.32%) |
| **Melanoma** | 24 (1.69%) | 144 (10.15%) | 362 (25.51%) | 562 (39.61%) | 327 (23.04%) |
| **Mesothelioma** | 7 (3.54%) | 33 (16.67%) | 57 (28.79%) | 62 (31.31%) | 39 (19.70%) |
| **Brain and CNS** | 16 (4.05%) | 39 (9.87%) | 126 (31.90%) | 137 (34.68%) | 77 (19.49%) |
| **Thyroid** | 2 (0.97%) | 27 (13.11%) | 58 (28.16%) | 75 (36.41%) | 44 (21.36%) |

*Brain and CNS, brain and central nervous system cancers.*

**Supplementary Table S5. The number of participants with different healthy lifestyle scores (United Kingdom,2006–2016).**

| **Participants** | **Healthy lifestyle scores** | | | |
| --- | --- | --- | --- | --- |
|  | **0-1** | **2** | **3** | **4** |
| **All participants included**  **in this study** | 30299 (7.97%) | 103832 (27.32%) | 155305 (40.87%) | 90606 (23.84%) |
| **Overall cancer** | 2372 (9.64%) | 6911 (28.07%) | 9755 (39.63%) | 5580 (22.67%) |
| **Head and neck** | 142 (23.13%) | 169 (27.52%) | 199 (32.41%) | 104 (16.94%) |
| **Esophagus** | 52 (12.50%) | 140 (33.65%) | 164 (39.42%) | 60 (14.42%) |
| **Stomach** | 35 (12.07%) | 78 (26.90%) | 111 (38.28%) | 66 (22.76%) |
| **Colorectal** | 238 (8.76%) | 821 (30.21%) | 1044 (38.41%) | 615 (22.63%) |
| **Liver** | 34 (13.33%) | 68 (26.67%) | 103 (40.39%) | 50 (19.61%) |
| **Pancreas** | 60 (12.30%) | 163 (33.40%) | 162 (33.20%) | 103 (21.11%) |
| **Breast** | 287 (5.95%) | 1185 (24.57%) | 2012 (41.72%) | 1339 (27.76%) |
| **Corpus uteri** | 26 (3.80%) | 150 (21.93%) | 314 (45.91%) | 194 (28.36%) |
| **Ovary** | 19 (4.03%) | 100 (21.23%) | 212 (45.01%) | 140 (29.72%) |
| **Prostate** | 453 (9.37%) | 1471 (30.42%) | 1975 (40.85%) | 936 (19.36%) |
| **Kidney** | 62 (9.48%) | 193 (29.51%) | 253 (38.69%) | 146 (22.32%) |
| **Bladder** | 122 (11.82%) | 307 (29.75%) | 395 (38.28%) | 208 (20.16%) |
| **Non-Hodgkin lymphoma** | 77 (7.75%) | 254 (25.58%) | 409 (41.19%) | 253 (25.48%) |
| **Multiple myeloma** | 30 (7.79%) | 103 (26.75%) | 150 (38.96%) | 102 (26.49%) |
| **Leukemia** | 48 (7.38%) | 175 (26.92%) | 283 (43.54%) | 144 (22.15%) |
| **Lung** | 372 (22.93%) | 533 (32.86%) | 483 (29.78%) | 234 (14.43%) |
| **Melanoma** | 103 (7.26%) | 375 (26.43%) | 582 (41.01%) | 359 (25.30%) |
| **Mesothelioma** | 18 (9.09%) | 60 (30.30%) | 83 (41.92%) | 37 (18.69%) |
| **Brain and CNS** | 37 (9.37%) | 113 (28.61%) | 162 (41.01%) | 83 (21.01%) |
| **Thyroid** | 12 (5.83%) | 45 (21.84%) | 89 (43.20%) | 60 (29.13%) |

*Brain and CNS, brain and central nervous system cancers.*

**Supplementary Table S6. The association of sleep patterns with overall and 20 types of cancers (United Kingdom,2006–2016).**

| **Type of cancer** | **Cases** | **Model 1** | | **Model 2** | | **Model 3** | | |
| --- | --- | --- | --- | --- | --- | --- | --- | --- |
|  |  | **HR(95%CI)** | ***P* value** | **HR(95%CI)** | ***P* value** | **HR(95%CI)** | ***P* value** | **FDR-adjusted *P*** |
| **Head and neck** | 614 | 0.87 (0.74,1.02) | 0.093 | 0.91 (0.77,1.06) | 0.226 | 0.97 (0.83,1.14) | 0.736 | 0.814 |
| **Esophagus** | 416 | **0.77 (0.63,0.93)** | **0.008** | **0.82 (0.67,0.99)** | **0.041** | 0.84 (0.69,1.02) | 0.078 | 0.182 |
| **Stomach** | 290 | 0.87 (0.69,1.10) | 0.248 | 0.96 (0.76,1.21) | 0.708 | 0.97 (0.76,1.22) | 0.775 | 0.814 |
| **Colorectal** | 2718 | **0.89 (0.83,0.96)** | **0.003** | **0.91 (0.84,0.98)** | **0.017** | **0.92 (0.85,0.99)** | **0.036** | 0.095 |
| **Liver** | 255 | **0.64 (0.50,0.82)** | **<0.001** | **0.72 (0.56,0.92)** | **0.010** | **0.73 (0.57,0.94)** | **0.015** | 0.077 |
| **Pancreas** | 488 | 0.96 (0.80,1.15) | 0.672 | 1.00 (0.84,1.20) | 0.973 | 1.04 (0.87,1.25) | 0.664 | 0.814 |
| **Breast** | 4823 | 0.95 (0.89,1.00) | 0.060 | 0.96 (0.90,1.02) | 0.163 | 0.97 (0.91,1.03) | 0.272 | 0.407 |
| **Corpus uteri** | 684 | **0.85 (0.73,0.99)** | **0.035** | 0.94 (0.80,1.09) | 0.411 | 0.93 (0.80,1.09) | 0.359 | 0.503 |
| **Ovary** | 471 | **1.25 (1.03,1.51)** | **0.022** | **1.26 (1.04,1.53)** | **0.020** | **1.25 (1.03,1.52)** | **0.024** | 0.089 |
| **Prostate** | 4835 | 0.97 (0.92,1.03) | 0.339 | 0.96 (0.90,1.01) | 0.133 | 0.96 (0.90,1.01) | 0.118 | 0.233 |
| **Kidney** | 654 | 0.92 (0.78,1.07) | 0.261 | 0.99 (0.85,1.16) | 0.912 | 1.00 (0.85,1.17) | 0.985 | 0.985 |
| **Bladder** | 1032 | **0.77 (0.68,0.86)** | **<0.001** | **0.81 (0.71,0.92)** | **<0.001** | **0.82 (0.73,0.93)** | **0.002** | **0.021** |
| **Non-Hodgkin lymphoma** | 993 | 0.97 (0.86,1.10) | 0.678 | 0.99 (0.87,1.12) | 0.825 | 0.98 (0.86,1.11) | 0.765 | 0.814 |
| **Multiple myeloma** | 385 | **0.79 (0.65,0.97)** | **0.021** | **0.79 (0.65,0.97)** | **0.025** | **0.79 (0.65,0.97)** | **0.026** | 0.089 |
| **Leukemia** | 650 | 0.89 (0.76,1.04) | 0.131 | 0.89 (0.76,1.04) | 0.151 | 0.89 (0.76,1.04) | 0.133 | 0.233 |
| **Lung** | 1622 | **0.75 (0.68,0.83)** | **<0.001** | **0.82 (0.74,0.91)** | **<0.001** | **0.90 (0.81,0.99)** | **0.030** | 0.089 |
| **Melanoma** | 1419 | **1.21 (1.09,1.35)** | **<0.001** | **1.18 (1.06,1.31)** | **0.003** | **1.17 (1.05,1.31)** | **0.004** | **0.028** |
| **Mesothelioma** | 198 | 0.79 (0.60,1.04) | 0.095 | 0.80 (0.60,1.06) | 0.116 | 0.80 (0.60,1.06) | 0.125 | 0.233 |
| **Brain and CNS** | 395 | 0.87 (0.72,1.07) | 0.183 | 0.89 (0.72,1.08) | 0.237 | 0.89 (0.73,1.09) | 0.260 | 0.407 |
| **Thyroid** | 206 | 0.93 (0.71,1.23) | 0.633 | 0.94 (0.71,1.24) | 0.651 | 0.93 (0.70,1.23) | 0.589 | 0.773 |
| **Overall cancer** | 24618 | **0.92 (0.90,0.95)** | **<0.001** | **0.94 (0.92,0.97)** | **<0.001** | **0.95 (0.93,0.98)** | **<0.001** | **0.006** |

*Brain and CNS, brain and central nervous system cancers. The reference group was the group with unhealthy sleep pattern. Model 1 was adjusted for sex and age. Model 2 was adjusted for sex, age, the Townsend Deprivation Index, ethnicity, sedentary time, employment, education, and BMI**. Model 3 was adjusted for sex, age, the Townsend Deprivation Index, ethnicity, sedentary time, employment, education, BMI,* *and lifestyle pattern. FDR, false-discovery rate. Bold values provided* P *value <0.05.*

**Supplementary Table S7. The association of lifestyle patterns with overall and 20 types of cancers (United Kingdom,2006–2016).**

| **Type of cancer** | **Lifestyle** | **Model 1** | | **Model 2** | | **Model 3** | | |
| --- | --- | --- | --- | --- | --- | --- | --- | --- |
|  |  | **HR(95%CI)** | ***P* value** | **HR(95%CI)** | ***P* value** | **HR(95%CI)** | ***P* value** | **FDR-adjusted *P*** |
| **Head and neck** | Intermediate | **0.36(0.29,0.45)** | **<0.001** | **0.39(0.31,0.49)** | **<0.001** | **0.39(0.31,0.49)** | **<0.001** | **<0.001** |
|  | Healthy | **0.29(0.24,0.36)** | **<0.001** | **0.32(0.26,0.39)** | **<0.001** | **0.32(0.26,0.39)** | **<0.001** | **<0.001** |
| **Esophagus** | Intermediate | 0.80(0.59,1.11) | 0.182 | 0.85(0.61,1.17) | 0.309 | 0.86(0.62,1.18) | 0.343 | 0.721 |
|  | Healthy | **0.57(0.42,0.78)** | **<0.001** | **0.63(0.46,0.85)** | **0.003** | **0.64(0.47,0.87)** | **0.005** | **0.020** |
| **Stomach** | Intermediate | **0.66(0.44,0.99)** | **0.042** | 0.73(0.49,1.10) | 0.134 | 0.74(0.49,1.10) | 0.138 | 0.321 |
|  | Healthy | **0.66(0.46,0.95)** | **0.025** | 0.75(0.51,1.09) | 0.130 | 0.75(0.52,1.10) | 0.137 | 0.321 |
| **Colorectal** | Intermediate | 0.98(0.85,1.14) | 0.826 | 1.00(0.86,1.15) | 0.973 | 1.00(0.87,1.16) | 0.968 | 0.968 |
|  | Healthy | **0.83(0.72,0.95)** | **0.006** | **0.85(0.74,0.97)** | **0.019** | **0.86(0.74,0.98)** | **0.028** | 0.091 |
| **Liver** | Intermediate | **0.58(0.39,0.88)** | **0.011** | **0.63(0.42,0.96)** | **0.032** | **0.65(0.43,0.98)** | **0.041** | 0.115 |
|  | Healthy | **0.56(0.39,0.82)** | **0.003** | **0.62(0.42,0.92)** | **0.016** | **0.65(0.44,0.95)** | **0.028** | 0.091 |
| **Pancreas** | Intermediate | 0.75(0.56,1.02) | 0.063 | 0.79(0.59,1.07) | 0.131 | 0.79(0.58,1.07) | 0.126 | 0.321 |
|  | Healthy | **0.49(0.37,0.66)** | **<0.001** | **0.53(0.40,0.71)** | **<0.001** | **0.53(0.40,0.71)** | **<0.001** | **<0.001** |
| **Breast** | Intermediate | **0.87(0.77,0.99)** | **0.041** | **0.87(0.76,0.99)** | **0.034** | **0.87(0.77,0.99)** | **0.038** | 0.114 |
|  | Healthy | **0.80(0.71,0.90)** | **<0.001** | **0.80(0.71,0.91)** | **<0.001** | **0.81(0.71,0.91)** | **<0.001** | **0.003** |
| **Corpus uteri** | Intermediate | 1.16(0.77,1.76) | 0.481 | 1.11(0.73,1.68) | 0.637 | 1.11(0.73,1.69) | 0.620 | 0.867 |
|  | Healthy | 1.21(0.81,1.79) | 0.347 | 1.21(0.82,1.80) | 0.337 | 1.23(0.82,1.82) | 0.315 | 0.696 |
| **Ovary** | Intermediate | 1.08(0.66,1.76) | 0.766 | 1.06(0.65,1.74) | 0.813 | 1.04(0.64,1.71) | 0.869 | 0.967 |
|  | Healthy | 1.18(0.75,1.88) | 0.474 | 1.18(0.74,1.87) | 0.491 | 1.14(0.72,1.82) | 0.578 | 0.867 |
| **Prostate** | Intermediate | 1.05(0.95,1.17) | 0.327 | 1.03(0.93,1.15) | 0.532 | 1.04(0.93,1.15) | 0.497 | 0.848 |
|  | Healthy | 1.06(0.96,1.18) | 0.219 | 1.04(0.94,1.15) | 0.444 | 1.05(0.95,1.16) | 0.383 | 0.732 |
| **Kidney** | Intermediate | 0.91(0.69,1.22) | 0.538 | 0.96(0.71,1.28) | 0.763 | 0.96(0.71,1.28) | 0.764 | 0.967 |
|  | Healthy | 0.81(0.62,1.07) | 0.137 | 0.88(0.67,1.16) | 0.361 | 0.88(0.67,1.16) | 0.363 | 0.726 |
| **Bladder** | Intermediate | **0.75(0.61,0.92)** | **0.007** | **0.77(0.62,0.95)** | **0.014** | **0.78(0.63,0.96)** | **0.019** | 0.073 |
|  | Healthy | **0.65(0.53,0.79)** | **<0.001** | **0.69(0.56,0.84)** | **<0.001** | **0.70(0.58,0.86)** | **<0.001** | **0.003** |
| **Non-Hodgkin lymphoma** | Intermediate | 0.94(0.73,1.22) | 0.644 | 0.98(0.75,1.27) | 0.860 | 0.98(0.76,1.27) | 0.868 | 0.967 |
|  | Healthy | 1.02(0.80,1.29) | 0.880 | 1.06(0.83,1.36) | 0.619 | 1.07(0.84,1.36) | 0.606 | 0.867 |
| **Multiple myeloma** | Intermediate | 0.97(0.64,1.46) | 0.879 | 0.96(0.64,1.45) | 0.862 | 0.98(0.65,1.47) | 0.922 | 0.967 |
|  | Healthy | 0.97(0.66,1.43) | 0.891 | 0.96(0.65,1.41) | 0.825 | 0.99(0.67,1.45) | 0.944 | 0.967 |
| **Leukemia** | Intermediate | 1.05(0.77,1.45) | 0.743 | 1.06(0.77,1.46) | 0.738 | 1.06(0.77,1.47) | 0.701 | 0.920 |
|  | Healthy | 1.09(0.81,1.47) | 0.576 | 1.11(0.82,1.50) | 0.503 | 1.13(0.83,1.53) | 0.443 | 0.809 |
| **Lung** | Intermediate | **0.38(0.33,0.43)** | **<0.001** | **0.44(0.38,0.50)** | **<0.001** | **0.44(0.38,0.50)** | **<0.001** | **<0.001** |
|  | Healthy | **0.20(0.17,0.22)** | **<0.001** | **0.24(0.21,0.27)** | **<0.001** | **0.24(0.21,0.28)** | **<0.001** | **<0.001** |
| **Melanoma** | Intermediate | 1.05(0.84,1.30) | 0.687 | 1.04(0.83,1.29) | 0.739 | 1.03(0.82,1.28) | 0.817 | 0.967 |
|  | Healthy | 1.09(0.89,1.34) | 0.387 | 1.09(0.89,1.35) | 0.404 | 1.07(0.87,1.32) | 0.525 | 0.848 |
| **Mesothelioma** | Intermediate | 0.98(0.58,1.66) | 0.942 | 1.02(0.60,1.74) | 0.932 | 1.04(0.61,1.76) | 0.892 | 0.967 |
|  | Healthy | 0.88(0.53,1.44) | 0.601 | 0.93(0.56,1.53) | 0.767 | 0.95(0.58,1.57) | 0.846 | 0.967 |
| **Brain and CNS** | Intermediate | 0.91(0.62,1.31) | 0.602 | 0.91(0.63,1.33) | 0.634 | 0.92(0.63,1.34) | 0.664 | 0.900 |
|  | Healthy | 0.85(0.60,1.21) | 0.372 | 0.88(0.62,1.24) | 0.460 | 0.89(0.62,1.26) | 0.513 | 0.848 |
| **Thyroid** | Intermediate | 0.97(0.51,1.84) | 0.927 | 0.95(0.50,1.81) | 0.884 | 0.96(0.51,1.82) | 0.898 | 0.967 |
|  | Healthy | 1.21(0.67,2.19) | 0.527 | 1.18(0.65,2.14) | 0.594 | 1.19(0.65,2.16) | 0.570 | 0.867 |
| **Overall cancer** | Intermediate | **0.82(0.78,0.86)** | **<0.001** | **0.83(0.79,0.87)** | **<0.001** | **0.84(0.80,0.88)** | **<0.001** | **<0.001** |
|  | Healthy | **0.74(0.71,0.77)** | **<0.001** | **0.76(0.73,0.80)** | **<0.001** | **0.77(0.73,0.80)** | **<0.001** | **<0.001** |

*Brain and CNS, brain and central nervous system cancers. The reference group was the group with unhealthy lifestyle pattern.* *Model 1 was adjusted for sex and age. Model 2 was adjusted for sex, age, the Townsend Deprivation Index, ethnicity, sedentary time, employment, education, and BMI. Model 3 was adjusted for sex, age, the Townsend Deprivation Index, ethnicity, sedentary time, employment, education, BMI, and sleep pattern. FDR, false-discovery rate. Bold values provided* P *value <0.05.*

**Supplementary Table S8. The association of sleep patterns with overall and 20 types of cancers after excluding cancers occurring within the first 2 years after the baseline (n = 374,038) (United Kingdom,2006–2016).**

| **Type of cancer** | **Cases** | **Model 1** | ***P* value** | **Model 2** | ***P* value** | **Model 3** | ***P* value** |
| --- | --- | --- | --- | --- | --- | --- | --- |
| **Head and neck** | 465 | 0.90(0.75,1.08) | 0.244 | 0.93(0.77,1.12) | 0.442 | 1.00(0.83,1.21) | 0.993 |
| **Esophagus** | 319 | **0.80(0.64,0.99)** | **0.043** | 0.85(0.68,1.06) | 0.159 | 0.87(0.70,1.09) | 0.236 |
| **Stomach** | 226 | 0.85(0.65,1.10) | 0.212 | 0.92(0.71,1.21) | 0.559 | 0.93(0.71,1.21) | 0.577 |
| **Colorectal** | 2023 | **0.87(0.80,0.95)** | **0.003** | **0.90(0.82,0.98)** | **0.020** | **0.91(0.83,0.99)** | **0.038** |
| **Liver** | 204 | **0.67(0.51,0.89)** | **0.005** | 0.77(0.58,1.02) | 0.070 | 0.79(0.59,1.04) | 0.092 |
| **Pancreas** | 396 | 0.97(0.80,1.19) | 0.778 | 1.02(0.84,1.25) | 0.817 | 1.06(0.86,1.29) | 0.602 |
| **Breast** | 3558 | 0.96(0.90,1.03) | 0.216 | 0.98(0.91,1.04) | 0.486 | 0.98(0.92,1.05) | 0.648 |
| **Corpus Uteri** | 503 | **0.81(0.68,0.96)** | **0.017** | 0.89(0.74,1.06) | 0.195 | 0.88(0.73,1.05) | 0.157 |
| **Ovary** | 344 | 1.23(0.99,1.54) | 0.064 | **1.26(1.00,1.58)** | **0.046** | 1.25(1.00,1.57) | 0.052 |
| **Prostate** | 3669 | 0.98(0.91,1.04) | 0.464 | 0.96(0.90,1.02) | 0.195 | 0.95(0.89,1.02) | 0.149 |
| **Kidney** | 525 | 0.94(0.79,1.11) | 0.465 | 1.02(0.85,1.21) | 0.847 | 1.03(0.86,1.23) | 0.750 |
| **Bladder** | 788 | **0.77(0.67,0.89)** | **<0.001** | **0.81(0.70,0.93)** | **0.004** | **0.83(0.72,0.95)** | **0.008** |
| **Non-Hodgkin lymphoma** | 751 | 0.92(0.79,1.06) | 0.244 | 0.93(0.81,1.08) | 0.367 | 0.93(0.80,1.07) | 0.313 |
| **Multiple myeloma** | 294 | 0.94(0.75,1.19) | 0.619 | 0.95(0.75,1.20) | 0.658 | 0.96(0.76,1.21) | 0.720 |
| **Leukemia** | 507 | 0.89(0.74,1.05) | 0.173 | 0.88(0.74,1.06) | 0.173 | 0.88(0.74,1.05) | 0.169 |
| **Lung** | 1279 | **0.74(0.67,0.83)** | **<0.001** | **0.81(0.73,0.91)** | **<0.001** | **0.89(0.80,1.00)** | **0.043** |
| **Melanoma** | 1066 | **1.18(1.05,1.34)** | **0.007** | **1.15(1.01,1.30)** | **0.032** | **1.14(1.01,1.30)** | **0.037** |
| **Mesothelioma** | 150 | 0.73(0.53,1.01) | 0.059 | 0.74(0.54,1.02) | 0.069 | 0.75(0.54,1.03) | 0.079 |
| **Brain and CNS** | 295 | 0.84(0.67,1.06) | 0.136 | 0.85(0.67,1.07) | 0.173 | 0.85(0.67,1.07) | 0.168 |
| **Thyroid** | 149 | 0.99(0.71,1.37) | 0.939 | 1.01(0.72,1.40) | 0.973 | 0.99(0.71,1.38) | 0.953 |
| **Overall cancer** | 18613 | **0.92(0.89,0.95)** | **<0.001** | **0.94(0.91,0.97)** | **<0.001** | **0.95(0.92,0.98)** | **0.001** |

*Brain and CNS, brain and central nervous system cancers. The reference group was the group of unhealthy sleep pattern. Model 1 was adjusted for sex and age. Model 2 was adjusted for sex, age, the Townsend Deprivation Index, ethnicity, sedentary time, employment, education, and BMI. Model 3 was adjusted for sex, age, the Townsend Deprivation Index, ethnicity, sedentary time, employment, education, BMI, and lifestyle pattern. Bold values provided* P *value <0.05.*

**Supplementary Table S9. The association of intermediate and healthy lifestyle patterns with overall and 20 types of cancers after excluding cancers occurring within the first 2 years after the baseline (n = 374,038) (United Kingdom,2006–2016).**

| **Type of cancer** | **Lifestyle** | **Model 1** | ***P* value** | **Model 2** | ***P* value** | **Model 3** | ***P* value** |
| --- | --- | --- | --- | --- | --- | --- | --- |
| **Head and neck** | Intermediate | **0.34(0.26,0.44)** | **<0.001** | **0.36(0.28,0.47)** | **<0.001** | **0.36(0.28,0.47)** | **<0.001** |
|  | Healthy | **0.28(0.22,0.36)** | **<0.001** | **0.31(0.24,0.39)** | **<0.001** | **0.31(0.24,0.39)** | **<0.001** |
| **Esophagus** | Intermediate | 0.85(0.59,1.23) | 0.389 | 0.89(0.61,1.29) | 0.539 | 0.90(0.62,1.30) | 0.570 |
|  | Healthy | **0.61(0.43,0.87)** | **0.006** | **0.66(0.46,0.95)** | **0.025** | **0.68(0.47,0.97)** | **0.032** |
| **Stomach** | Intermediate | **0.55(0.35,0.87)** | **0.011** | **0.61(0.38,0.97)** | **0.038** | **0.61(0.38,0.98)** | **0.040** |
|  | Healthy | 0.67(0.45,1.01) | 0.058 | 0.76(0.50,1.16) | 0.204 | 0.77(0.50,1.17) | 0.222 |
| **Colorectal** | Intermediate | 1.01(0.85,1.19) | 0.927 | 1.03(0.87,1.22) | 0.764 | 1.03(0.87,1.22) | 0.708 |
|  | Healthy | **0.83(0.71,0.98)** | **0.027** | 0.86(0.73,1.01) | 0.061 | 0.87(0.74,1.02) | 0.084 |
| **Liver** | Intermediate | **0.56(0.35,0.88)** | **0.012** | **0.61(0.38,0.97)** | **0.038** | **0.62(0.39,0.99)** | **0.045** |
|  | Healthy | **0.56(0.37,0.85)** | **0.006** | **0.63(0.41,0.96)** | **0.033** | **0.65(0.42,0.99)** | **0.047** |
| **Pancreas** | Intermediate | 0.73(0.52,1.03) | 0.070 | 0.78(0.56,1.10) | 0.163 | 0.78(0.55,1.10) | 0.157 |
|  | Healthy | **0.54(0.39,0.74)** | **<0.001** | **0.59(0.42,0.81)** | **0.001** | **0.58(0.42,0.81)** | **0.001** |
| **Breast** | Intermediate | 0.89(0.76,1.03) | 0.114 | 0.87(0.75,1.02) | 0.080 | 0.87(0.75,1.02) | 0.083 |
|  | Healthy | **0.82(0.71,0.94)** | **0.005** | **0.81(0.70,0.94)** | **0.004** | **0.81(0.71,0.94)** | **0.005** |
| **Corpus uteri** | Intermediate | 1.38(0.82,2.33) | 0.229 | 1.32(0.78,2.23) | 0.301 | 1.33(0.79,2.25) | 0.285 |
|  | Healthy | 1.45(0.88,2.40) | 0.143 | 1.46(0.88,2.41) | 0.141 | 1.48(0.90,2.46) | 0.124 |
| **Ovary** | Intermediate | 1.08(0.61,1.91) | 0.794 | 1.08(0.61,1.91) | 0.801 | 1.06(0.60,1.88) | 0.849 |
|  | Healthy | 1.17(0.68,2.00) | 0.575 | 1.18(0.68,2.02) | 0.556 | 1.14(0.66,1.96) | 0.635 |
| **Prostate** | Intermediate | 1.06(0.94,1.19) | 0.377 | 1.04(0.92,1.17) | 0.555 | 1.04(0.92,1.18) | 0.522 |
|  | Healthy | 1.11(0.99,1.25) | 0.065 | 1.09(0.97,1.22) | 0.146 | 1.10(0.98,1.23) | 0.121 |
| **Kidney** | Intermediate | 0.94(0.68,1.29) | 0.704 | 0.95(0.69,1.30) | 0.742 | 0.95(0.69,1.30) | 0.733 |
|  | Healthy | 0.80(0.59,1.08) | 0.146 | 0.83(0.61,1.13) | 0.231 | 0.83(0.61,1.12) | 0.224 |
| **Bladder** | Intermediate | **0.73(0.57,0.93)** | **0.010** | **0.75(0.59,0.95)** | **0.019** | **0.76(0.60,0.97)** | **0.025** |
|  | Healthy | **0.63(0.51,0.79)** | **<0.001** | **0.66(0.53,0.83)** | **<0.001** | **0.68(0.54,0.85)** | **<0.001** |
| **Non-Hodgkin lymphoma** | Intermediate | 0.98(0.73,1.32) | 0.899 | 1.04(0.77,1.40) | 0.817 | 1.04(0.77,1.41) | 0.791 |
|  | Healthy | 1.07(0.81,1.42) | 0.628 | 1.14(0.86,1.52) | 0.367 | 1.15(0.87,1.53) | 0.333 |
| **Multiple myeloma** | Intermediate | 0.87(0.56,1.36) | 0.542 | 0.87(0.56,1.35) | 0.525 | 0.87(0.56,1.35) | 0.534 |
|  | Healthy | 0.82(0.54,1.24) | 0.345 | 0.80(0.53,1.22) | 0.297 | 0.80(0.53,1.22) | 0.310 |
| **Leukemia** | Intermediate | 1.06(0.74,1.52) | 0.739 | 1.05(0.73,1.50) | 0.792 | 1.06(0.74,1.52) | 0.757 |
|  | Healthy | 1.04(0.74,1.46) | 0.835 | 1.04(0.74,1.46) | 0.832 | 1.05(0.75,1.48) | 0.763 |
| **Lung** | Intermediate | **0.37(0.31,0.42)** | **<0.001** | **0.42(0.36,0.49)** | **<0.001** | **0.42(0.36,0.49)** | **<0.001** |
|  | Healthy | **0.19(0.17,0.22)** | **<0.001** | **0.23(0.20,0.27)** | **<0.001** | **0.24(0.20,0.28)** | **<0.001** |
| **Melanoma** | Intermediate | 0.98(0.77,1.26) | 0.883 | 0.98(0.76,1.26) | 0.870 | 0.97(0.76,1.25) | 0.812 |
|  | Healthy | 1.05(0.83,1.32) | 0.671 | 1.05(0.83,1.33) | 0.668 | 1.03(0.82,1.31) | 0.777 |
| **Mesothelioma** | Intermediate | 0.81(0.45,1.43) | 0.463 | 0.84(0.47,1.50) | 0.562 | 0.86(0.48,1.53) | 0.604 |
|  | Healthy | 0.73(0.43,1.25) | 0.253 | 0.78(0.45,1.33) | 0.361 | 0.80(0.47,1.38) | 0.432 |
| **Brain and CNS** | Intermediate | 0.90(0.58,1.41) | 0.658 | 0.91(0.58,1.41) | 0.663 | 0.92(0.59,1.43) | 0.700 |
|  | Healthy | 0.95(0.63,1.44) | 0.810 | 0.97(0.64,1.48) | 0.896 | 0.99(0.65,1.51) | 0.975 |
| **Thyroid** | Intermediate | 0.90(0.43,1.89) | 0.776 | 0.90(0.43,1.89) | 0.773 | 0.90(0.43,1.89) | 0.775 |
|  | Healthy | 1.20(0.60,2.38) | 0.604 | 1.19(0.60,2.38) | 0.619 | 1.19(0.60,2.38) | 0.617 |
| **Overall cancer** | Intermediate | **0.81(0.76,0.85)** | **<0.001** | **0.82(0.78,0.87)** | **<0.001** | **0.82(0.78,0.87)** | **<0.001** |
|  | Healthy | **0.74(0.71,0.78)** | **<0.001** | **0.76(0.72,0.80)** | **<0.001** | **0.77(0.73,0.81)** | **<0.001** |

*Brain and CNS, brain and central nervous system cancers. The reference group was the group of unhealthy lifestyle pattern. Model 1 was adjusted for sex and age. Model 2 was adjusted for sex, age, the Townsend Deprivation Index, ethnicity, sedentary time, employment, education, and BMI. Model 3 was adjusted for sex, age, the Townsend Deprivation Index, ethnicity, sedentary time, employment, education, BMI, and sleep pattern. Bold values provided* P *value <0.05.*

**Supplementary Table S10. The association of sleep patterns with overall and 20 types of cancers stratified by sex (United Kingdom,2006–2016).**

| **Type of cancer** | **Female** | | | **Male** | | |
| --- | --- | --- | --- | --- | --- | --- |
|  | **Cases** | **HR(95%CI)** | ***P* value** | **Cases** | **HR(95%CI)** | ***P* value** |
| **Head and neck** | 207 | 1.02(0.77,1.36) | 0.886 | 407 | 0.95(0.78,1.15) | 0.583 |
| **Esophagus** | 105 | 0.94(0.63,1.39) | 0.752 | 311 | 0.80(0.64,1.01) | 0.058 |
| **Stomach** | 87 | 0.91(0.59,1.41) | 0.669 | 203 | 0.99(0.75,1.31) | 0.931 |
| **Colorectal** | 1165 | 0.99(0.88,1.11) | 0.857 | 1553 | **0.87(0.79,0.96)** | **0.006** |
| **Liver** | 93 | 0.70(0.46,1.05) | 0.087 | 162 | 0.75(0.55,1.03) | 0.073 |
| **Pancreas** | 214 | 0.96(0.73,1.26) | 0.761 | 274 | 1.11(0.87,1.42) | 0.395 |
| **Breast** | 4823 | 0.97(0.91,1.03) | 0.272 | - | - | - |
| **Corpus uteri** | 684 | 0.93(0.80,1.09) | 0.359 | - | - | - |
| **Ovary** | 471 | **1.25(1.03,1.52)** | **0.024** | - | - | - |
| **Prostate** | - | - | - | 4835 | 0.96(0.90,1.01) | 0.118 |
| **Kidney** | 235 | 1.04(0.80,1.35) | 0.798 | 419 | 0.98(0.81,1.19) | 0.853 |
| **Bladder** | 265 | 0.86(0.67,1.10) | 0.234 | 767 | **0.81(0.70,0.94)** | **0.004** |
| **Non-Hodgkin lymphoma** | 450 | 0.84(0.70,1.02) | 0.076 | 543 | 1.11(0.94,1.33) | 0.219 |
| **Multiple myeloma** | 175 | 0.90(0.66,1.21) | 0.479 | 210 | **0.72(0.55,0.95)** | **0.020** |
| **Leukemia** | 260 | 0.86(0.67,1.10) | 0.237 | 390 | 0.91(0.74,1.11) | 0.335 |
| **Lung** | 757 | **0.82(0.71,0.95)** | **0.007** | 865 | 0.97(0.84,1.11) | 0.618 |
| **Melanoma** | 708 | 1.10(0.94,1.29) | 0.217 | 711 | **1.23(1.06,1.44)** | **0.007** |
| **Mesothelioma** | 36 | 0.60(0.31,1.17) | 0.137 | 162 | 0.85(0.62,1.17) | 0.319 |
| **Brain and CNS** | 154 | 0.97(0.70,1.34) | 0.841 | 241 | 0.85(0.66,1.10) | 0.210 |
| **Thyroid** | 153 | 0.80(0.58,1.11) | 0.189 | 53 | 1.39(0.78,2.45) | 0.261 |
| **Overall cancer** | 11788 | **0.96(0.92,0.99)** | **0.016** | 12830 | **0.94(0.91,0.98)** | **0.001** |

*Brain and CNS, brain and central nervous system cancers. The reference group was the group with unhealthy sleep pattern. Model was adjusted for age, the Townsend Deprivation Index, ethnicity, sedentary time, employment, education, BMI, and lifestyle pattern. Bold values provided* P *value <0.05.*

**Supplementary Table S11. The association of lifestyle patterns with overall and 20 types of cancers stratified by sex (United Kingdom,2006–2016).**

| **Type of cancer** | **Lifestyle** | **Female** | | | **Male** | | |
| --- | --- | --- | --- | --- | --- | --- | --- |
|  |  | **Cases(n,%)** | **HR(95%CI)** | ***P* value** | **Cases(n,%)** | **HR(95%CI)** | ***P* value** |
| **Head and neck** | Intermediate | 50(24.15) | **0.43(0.27,0.70)** | **<0.001** | 119(29.24) | **0.38(0.30,0.50)** | **<0.001** |
|  | Healthy | 131(63.29) | **0.36(0.23,0.56)** | **<0.001** | 172(42.26) | **0.30(0.24,0.39)** | **<0.001** |
| **Esophagus** | Intermediate | 28(26.67) | 0.59(0.29,1.22) | 0.157 | 112(36.01) | 0.92(0.65,1.32) | 0.661 |
|  | Healthy | 67(63.81) | **0.42(0.22,0.83)** | **0.013** | 157(50.48) | **0.70(0.50,0.99)** | **0.044** |
| **Stomach** | Intermediate | 12(13.79) | **0.34(0.14,0.85)** | **0.020** | 66(32.51) | 0.88(0.56,1.39) | 0.582 |
|  | Healthy | 67(77.01) | 0.61(0.29,1.30) | 0.202 | 110(54.19) | 0.77(0.50,1.18) | 0.231 |
| **Colorectal** | Intermediate | 276(23.69) | 0.87(0.66,1.15) | 0.334 | 545(35.09) | 1.07(0.90,1.27) | 0.468 |
|  | Healthy | 826(70.90) | 0.80(0.62,1.03) | 0.085 | 833(53.64) | 0.87(0.74,1.03) | 0.101 |
| **Liver** | Intermediate | 19(20.43) | 0.69(0.27,1.73) | 0.426 | 49(30.25) | 0.65(0.40,1.04) | 0.072 |
|  | Healthy | 68(73.12) | 0.80(0.34,1.87) | 0.610 | 85(52.47) | **0.60(0.39,0.94)** | **0.024** |
| **Pancreas** | Intermediate | 48(22.43) | **0.51(0.30,0.87)** | **0.013** | 115(41.97) | 0.97(0.67,1.40) | 0.874 |
|  | Healthy | 147(68.69) | **0.48(0.29,0.77)** | **0.003** | 118(43.07) | **0.53(0.36,0.76)** | **<0.001** |
| **Breast** | Intermediate | 1185(24.57) | **0.87(0.77,0.99)** | **0.038** | - | - | - |
|  | Healthy | 3351(69.48) | **0.81(0.71,0.91)** | **<0.001** | - | - | - |
| **Corpus uteri** | Intermediate | 150(21.93) | 1.11(0.73,1.69) | 0.620 | - | - | - |
|  | Healthy | 508(74.27) | 1.23(0.82,1.82) | 0.315 | - | - | - |
| **Ovary** | Intermediate | 100(21.23) | 1.04(0.64,1.71) | 0.869 | - | - | - |
|  | Healthy | 352(74.73) | 1.14(0.72,1.82) | 0.578 | - | - | - |
| **Prostate** | Intermediate | - | - | - | 1471(30.42) | 1.04(0.93,1.15) | 0.497 |
|  | Healthy | - | - | - | 2911(60.21) | 1.05(0.95,1.16) | 0.383 |
| **Kidney** | Intermediate | 58(24.68) | 0.63(0.37,1.06) | 0.079 | 135(32.22) | 1.11(0.78,1.57) | 0.576 |
|  | Healthy | 158(67.23) | **0.55(0.34,0.89)** | **0.015** | 241(57.52) | 1.05(0.75,1.47) | 0.770 |
| **Bladder** | Intermediate | 97(21.56) | 0.81(0.48,1.39) | 0.450 | 242(31.55) | **0.76(0.61,0.96)** | **0.022** |
|  | Healthy | 329(73.11) | 0.72(0.44,1.19) | 0.203 | 420(54.76) | **0.70(0.56,0.87)** | **0.001** |
| **Non-Hodgkin lymphoma** | Intermediate | 97(21.56) | 0.84(0.54,1.32) | 0.461 | 157(28.91) | 1.03(0.75,1.41) | 0.863 |
|  | Healthy | 329(73.11) | 0.90(0.59,1.37) | 0.626 | 333(61.33) | 1.15(0.85,1.54) | 0.367 |
| **Multiple myeloma** | Intermediate | 44(25.14) | 0.97(0.47,2.00) | 0.944 | 59(28.10) | 0.94(0.57,1.55) | 0.813 |
|  | Healthy | 122(69.71) | 0.83(0.42,1.64) | 0.587 | 130(61.90) | 1.09(0.68,1.73) | 0.723 |
| **Leukemia** | Intermediate | 47(18.08) | 0.73(0.40,1.36) | 0.324 | 128(32.82) | 1.23(0.84,1.79) | 0.279 |
|  | Healthy | 200(76.92) | 0.97(0.55,1.71) | 0.924 | 227(58.21) | 1.17(0.81,1.67) | 0.404 |
| **Lung** | Intermediate | 232(30.65) | **0.39(0.31,0.48)** | **<0.001** | 301(34.80) | **0.47(0.39,0.56)** | **<0.001** |
|  | Healthy | 392(51.78) | **0.21(0.17,0.26)** | **<0.001** | 325(37.57) | **0.27(0.22,0.32)** | **<0.001** |
| **Melanoma** | Intermediate | 161(22.74) | 1.06(0.72,1.55) | 0.784 | 214(30.10) | 1.00(0.77,1.32) | 0.982 |
|  | Healthy | 515(72.74) | 1.07(0.74,1.54) | 0.718 | 426(59.92) | 1.07(0.83,1.39) | 0.580 |
| **Mesothelioma** | Intermediate | 10(27.78) | 1.08(0.24,4.96) | 0.922 | 50(30.86) | 1.01(0.57,1.78) | 0.972 |
|  | Healthy | 24(66.67) | 0.80(0.19,3.44) | 0.763 | 96(59.26) | 0.97(0.57,1.65) | 0.905 |
| **Brain and CNS** | Intermediate | 30(19.48) | 0.63(0.31,1.30) | 0.212 | 83(34.44) | 1.06(0.69,1.65) | 0.783 |
|  | Healthy | 114(74.03) | 0.77(0.40,1.48) | 0.436 | 131(54.36) | 0.91(0.60,1.39) | 0.670 |
| **Thyroid** | Intermediate | 33(21.57) | 1.15(0.48,2.75) | 0.756 | 12(22.64) | 0.70(0.26,1.89) | 0.487 |
|  | Healthy | 114(74.51) | 1.29(0.56,2.94) | 0.551 | 35(66.04) | 1.11(0.46,2.68) | 0.810 |
| **Overall cancer** | Intermediate | 2813(23.86) | **0.75(0.70,0.82)** | **<0.001** | 4098(31.94) | **0.87(0.82,0.92)** | **<0.001** |
|  | Healthy | 8192(69.49) | **0.70(0.65,0.76)** | **<0.001** | 7143(55.67) | **0.79(0.75,0.84)** | **<0.001** |

*Brain and CNS, brain and central nervous system cancers. The reference group was the group with unhealthy lifestyle pattern. Model was adjusted for age, the Townsend Deprivation Index, ethnicity, sedentary time, employment, education, BMI, and sleep pattern. Bold values provided* P *value <0.05.*

**Supplementary Table S12. The association of the healthy sleep scores as a continuous variable with overall and 20 types of cancers (United Kingdom,2006–2016).**

| **Type of cancer** | **Model 1** | | **Model 2** | | **Model 3** | | **Model 3 (adjust data)** | |
| --- | --- | --- | --- | --- | --- | --- | --- | --- |
|  | **HR (95%CI)** | ***P* value** | **HR (95%CI)** | ***P* value** | **HR (95%CI)** | ***P* value** | **HR (95%CI)** | ***P* value** |
| **Head and neck** | 0.93(0.86,1.01) | 0.085 | 0.96(0.88,1.04) | 0.281 | 1.00(0.92,1.08) | 0.964 | 1.01(0.92,1.10) | 0.870 |
| **Esophagus** | **0.83(0.76,0.92)** | **<0.001** | **0.86(0.79,0.95)** | **0.003** | **0.88(0.80,0.97)** | **0.007** | 0.91(0.81,1.01) | 0.074 |
| **Stomach** | 0.91(0.82,1.02) | 0.122 | 0.96(0.86,1.08) | 0.513 | 0.97(0.86,1.09) | 0.581 | 0.95(0.83,1.08) | 0.457 |
| **Colorectal** | **0.96(0.93,1.00)** | **0.048** | 0.98(0.94,1.01) | 0.210 | 0.98(0.95,1.02) | 0.363 | 0.99(0.95,1.03) | 0.657 |
| **Liver** | **0.79(0.70,0.89)** | **<0.001** | **0.85(0.75,0.95)** | **0.006** | **0.85(0.76,0.96)** | **0.010** | **0.87(0.76,0.99)** | **0.035** |
| **Pancreas** | 0.96(0.88,1.04) | 0.327 | 0.98(0.90,1.07) | 0.698 | 1.00(0.92,1.10) | 0.929 | 1.01(0.91,1.11) | 0.856 |
| **Breast** | 0.97(0.95,1.00) | 0.070 | 0.98(0.96,1.01) | 0.231 | 0.99(0.96,1.02) | 0.402 | 1.00(0.97,1.04) | 0.919 |
| **Corpus uteri** | **0.91(0.85,0.98)** | **0.010** | 0.96(0.89,1.03) | 0.292 | 0.96(0.89,1.03) | 0.242 | 0.95(0.87,1.03) | 0.206 |
| **Ovary** | 1.07(0.98,1.17) | 0.125 | 1.08(0.98,1.18) | 0.121 | 1.07(0.98,1.17) | 0.144 | 1.07(0.96,1.19) | 0.228 |
| **Prostate** | 1.00(0.97,1.02) | 0.788 | 0.99(0.96,1.01) | 0.340 | 0.98(0.96,1.01) | 0.307 | 0.99(0.96,1.02) | 0.550 |
| **Kidney** | **0.93(0.86,1.00)** | **0.048** | 0.97(0.90,1.05) | 0.463 | 0.98(0.90,1.05) | 0.530 | 0.99(0.91,1.07) | 0.756 |
| **Bladder** | **0.87(0.82,0.93)** | **<0.001** | **0.90(0.85,0.96)** | **<0.001** | **0.91(0.86,0.97)** | **0.003** | **0.92(0.86,0.99)** | **0.026** |
| **Non-Hodgkin lymphoma** | 0.94(0.89,1.00) | 0.064 | 0.95(0.89,1.01) | 0.119 | 0.95(0.89,1.01) | 0.097 | 0.93(0.87,1.00) | 0.062 |
| **Multiple myeloma** | **0.89(0.81,0.98)** | **0.020** | **0.90(0.81,0.99)** | **0.029** | **0.90(0.81,0.99)** | **0.030** | 0.96(0.86,1.08) | 0.514 |
| **Leukemia** | 0.95(0.88,1.03) | 0.218 | 0.95(0.88,1.03) | 0.237 | 0.95(0.88,1.03) | 0.207 | 0.95(0.87,1.03) | 0.223 |
| **Lung** | **0.84(0.80,0.88)** | **<0.001** | **0.88(0.84,0.93)** | **<0.001** | **0.93(0.88,0.97)** | **0.002** | **0.94(0.89,0.99)** | **0.020** |
| **Melanoma** | **1.09(1.04,1.15)** | **<0.001** | **1.08(1.02,1.13)** | **0.008** | **1.07(1.02,1.13)** | **0.011** | 1.06(0.99,1.13) | 0.075 |
| **Mesothelioma** | 0.88(0.77,1.01) | 0.064 | 0.88(0.77,1.02) | 0.081 | 0.89(0.77,1.02) | 0.088 | 0.87(0.74,1.02) | 0.090 |
| **Brain and CNS** | 0.94(0.86,1.04) | 0.234 | 0.95(0.86,1.05) | 0.309 | 0.95(0.86,1.05) | 0.342 | 0.94(0.84,1.06) | 0.329 |
| **Thyroid** | 0.97(0.85,1.11) | 0.703 | 0.98(0.85,1.12) | 0.765 | 0.97(0.85,1.12) | 0.686 | 0.98(0.84,1.16) | 0.844 |
| **Overall cancer** | **0.96(0.95,0.97)** | **<0.001** | **0.97(0.96,0.98)** | **<0.001** | **0.98(0.96,0.99)** | **<0.001** | **0.98(0.97,1.00)** | **0.010** |

*Brain and CNS, brain and central nervous system cancers. Model 1 was adjusted for sex and age. Model 2 was adjusted for sex, age, the Townsend Deprivation Index, ethnicity, sedentary time, employment, education, and BMI. Model 3 was adjusted for* *sex, age, the Townsend Deprivation Index, ethnicity, sedentary time, employment, education, BMI, and lifestyle pattern**. Adjust data indicates the analysis was performed using data excluding the participants who were diagnosed cancer in the first two years of follow-up (n = 374,038). Bold values provided* P *value <0.05.*

**Supplementary Table S13. The association of healthy lifestyle scores as a continuous variable with overall and 20 types of cancers (United Kingdom,2006–2016).**

| **Type of cancer** | **Model 1** | | **Model 2** | | **Model 3** | | **Model 3 (adjust data)** | |
| --- | --- | --- | --- | --- | --- | --- | --- | --- |
|  | **HR (95%CI)** | ***P* value** | **HR (95%CI)** | ***P* value** | **HR (95%CI)** | ***P* value** | **HR (95%CI)** | ***P* value** |
| **Head and neck** | **0.36(0.29,0.45)** | **<0.001** | **0.39(0.31,0.49)** | **<0.001** | **0.39(0.31,0.49)** | **<0.001** | **0.67(0.60,0.74)** | **<0.001** |
| **Esophagus** | 0.80(0.58,1.10) | 0.178 | 0.84(0.61,1.16) | 0.301 | 0.85(0.62,1.18) | 0.334 | **0.82(0.72,0.93)** | **0.002** |
| **Stomach** | 0.66(0.44,0.99) | 0.042 | 0.73(0.49,1.10) | 0.136 | 0.74(0.49,1.10) | 0.139 | 1.01(0.87,1.18) | 0.872 |
| **Colorectal** | 0.98(0.85,1.14) | 0.826 | 1.00(0.86,1.15) | 0.974 | 1.00(0.87,1.16) | 0.966 | **0.93(0.88,0.98)** | **0.004** |
| **Liver** | **0.58(0.39,0.88)** | **0.011** | **0.63(0.42,0.96)** | **0.031** | **0.65(0.42,0.98)** | **0.040** | 0.89(0.76,1.04) | 0.154 |
| **Pancreas** | 0.75(0.56,1.02) | 0.063 | 0.79(0.59,1.07) | 0.132 | 0.79(0.58,1.07) | 0.127 | **0.83(0.74,0.92)** | **0.001** |
| **Breast** | **0.93(0.90,0.96)** | **<0.001** | **0.93(0.90,0.97)** | **<0.001** | **0.93(0.90,0.97)** | **<0.001** | **0.94(0.91,0.98)** | **0.002** |
| **Corpus uteri** | 0.99(0.91,1.08) | 0.829 | 1.01(0.92,1.10) | 0.860 | 1.01(0.92,1.11) | 0.812 | 1.05(0.95,1.17) | 0.361 |
| **Ovary** | 1.02(0.92,1.14) | 0.654 | 1.03(0.92,1.15) | 0.636 | 1.02(0.91,1.13) | 0.768 | 1.02(0.90,1.16) | 0.775 |
| **Prostate** | 1.02(0.98,1.05) | 0.339 | 1.01(0.98,1.04) | 0.620 | 1.01(0.98,1.04) | 0.534 | 1.03(1.00,1.07) | 0.084 |
| **Kidney** | 0.91(0.69,1.22) | 0.538 | 0.96(0.72,1.28) | 0.765 | 0.96(0.72,1.28) | 0.766 | 0.94(0.85,1.04) | 0.238 |
| **Bladder** | **0.75(0.61,0.92)** | **0.007** | **0.77(0.62,0.95)** | **0.014** | **0.78(0.63,0.96)** | **0.019** | **0.90(0.83,0.97)** | **0.007** |
| **Non-Hodgkin lymphoma** | 0.94(0.73,1.22) | 0.647 | 0.98(0.75,1.27) | 0.864 | 0.98(0.76,1.27) | 0.872 | 1.07(0.99,1.17) | 0.091 |
| **Multiple myeloma** | 0.97(0.65,1.46) | 0.884 | 0.97(0.64,1.45) | 0.868 | 0.98(0.65,1.48) | 0.930 | 0.96(0.84,1.10) | 0.547 |
| **Leukemia** | 1.05(0.77,1.45) | 0.749 | 1.05(0.77,1.45) | 0.745 | 1.06(0.77,1.47) | 0.708 | 0.98(0.88,1.08) | 0.645 |
| **Lung** | **0.38(0.33,0.43)** | **<0.001** | **0.44(0.38,0.50)** | **<0.001** | **0.44(0.38,0.50)** | **<0.001** | **0.57(0.54,0.61)** | **<0.001** |
| **Melanoma** | 1.05(0.84,1.30) | 0.684 | 1.04(0.83,1.30) | 0.734 | 1.03(0.82,1.28) | 0.811 | 1.02(0.95,1.09) | 0.572 |
| **Mesothelioma** | 0.98(0.58,1.66) | 0.939 | 1.02(0.60,1.73) | 0.936 | 1.04(0.61,1.76) | 0.896 | 0.95(0.79,1.14) | 0.568 |
| **Brain and CNS** | 0.90(0.62,1.31) | 0.599 | 0.91(0.63,1.33) | 0.630 | 0.92(0.63,1.34) | 0.660 | 1.00(0.88,1.14) | 0.992 |
| **Thyroid** | 0.97(0.51,1.84) | 0.928 | 0.95(0.50,1.81) | 0.886 | 0.96(0.51,1.82) | 0.901 | 1.08(0.89,1.31) | 0.415 |
| **Overall cancer** | **0.82(0.78,0.86)** | **<0.001** | **0.83(0.79,0.87)** | **<0.001** | **0.84(0.80,0.88)** | **<0.001** | **0.92(0.91,0.94)** | **<0.001** |

*Brain and CNS, brain and central nervous system cancers. Model 1 was adjusted for sex and age. Model 2 was adjusted for sex, age, the Townsend Deprivation Index, ethnicity, sedentary time, employment, education, and BMI. Model 3 was adjusted for sex, age, the Townsend Deprivation Index, ethnicity, sedentary time, employment, education, BMI, and sleep pattern. Adjust data indicates the analysis was performed using data excluding the participants who were diagnosed cancer in the first two years of follow-up (n = 374,038). Bold values provided* P *value <0.05.*

**Supplementary Table S14. The joint association of sleep pattern and lifestyle pattern with overall cancer, liver cancer, bladder cancer, lung cancer and colorectal cancer after excluding cancers occurring within the first 2 years after the baseline (N = 374,038) (United Kingdom,2006–2016).**

| **Sleep pattern** | **Lifestyle pattern** | **Overall cancer** | | **Liver cancer** | | **Bladder cancer** | | **Lung cancer** | | **Colorectal cancer** | |
| --- | --- | --- | --- | --- | --- | --- | --- | --- | --- | --- | --- |
|  |  | **HR (95% CI)^a^** | ***P* value^a^** | **HR (95% CI)^a^** | ***P* value^a^** | **HR (95% CI)^a^** | ***P* value^a^** | **HR (95% CI)^a^** | ***P* value^a^** | **HR (95% CI)^a^** | ***P* value^a^** |
| Unhealthy | Unhealthy | - | - | - | - | - | - | - | - | - | - |
|  | Intermediate | **0.82(0.76,0.88)** | **<0.001** | 0.65(0.34,1.25) | 0.193 | 0.82(0.59,1.13) | 0.230 | **0.47(0.38,0.57)** | **<0.001** | 1.05(0.83,1.32) | 0.706 |
|  | Healthy | **0.78(0.72,0.83)** | **<0.001** | 0.90(0.50,1.61) | 0.712 | **0.73(0.54,0.99)** | **0.041** | **0.24(0.20,0.30)** | **<0.001** | 0.88(0.71,1.10) | 0.273 |
| Healthy | Unhealthy | 0.96(0.88,1.06) | 0.435 | 1.27(0.59,2.70) | 0.539 | 0.96(0.64,1.44) | 0.840 | 0.98(0.77,1.23) | 0.851 | 0.94(0.70,1.28) | 0.705 |
|  | Intermediate | **0.80(0.74,0.86)** | **<0.001** | 0.71(0.37,1.35) | 0.297 | **0.66(0.47,0.91)** | **0.012** | **0.37(0.30,0.46)** | **<0.001** | 0.96(0.76,1.21) | 0.716 |
|  | Healthy | **0.73(0.68,0.78)** | **<0.001** | **0.55(0.30,1.00)** | **0.050** | **0.59(0.44,0.80)** | **<0.001** | **0.22(0.18,0.27)** | **<0.001** | **0.80(0.64,0.99)** | **0.040** |

*^a^The hazard ratios (HRs), corresponding 95% confidence intervals (CIs) and* P *values were calculated using the Cox regression model adjusting for sex, age, Townsend Deprivation Index, ethnicity, sedentary time, employment, education, and BMI. Bold values provided* P *value <0.05.*

**
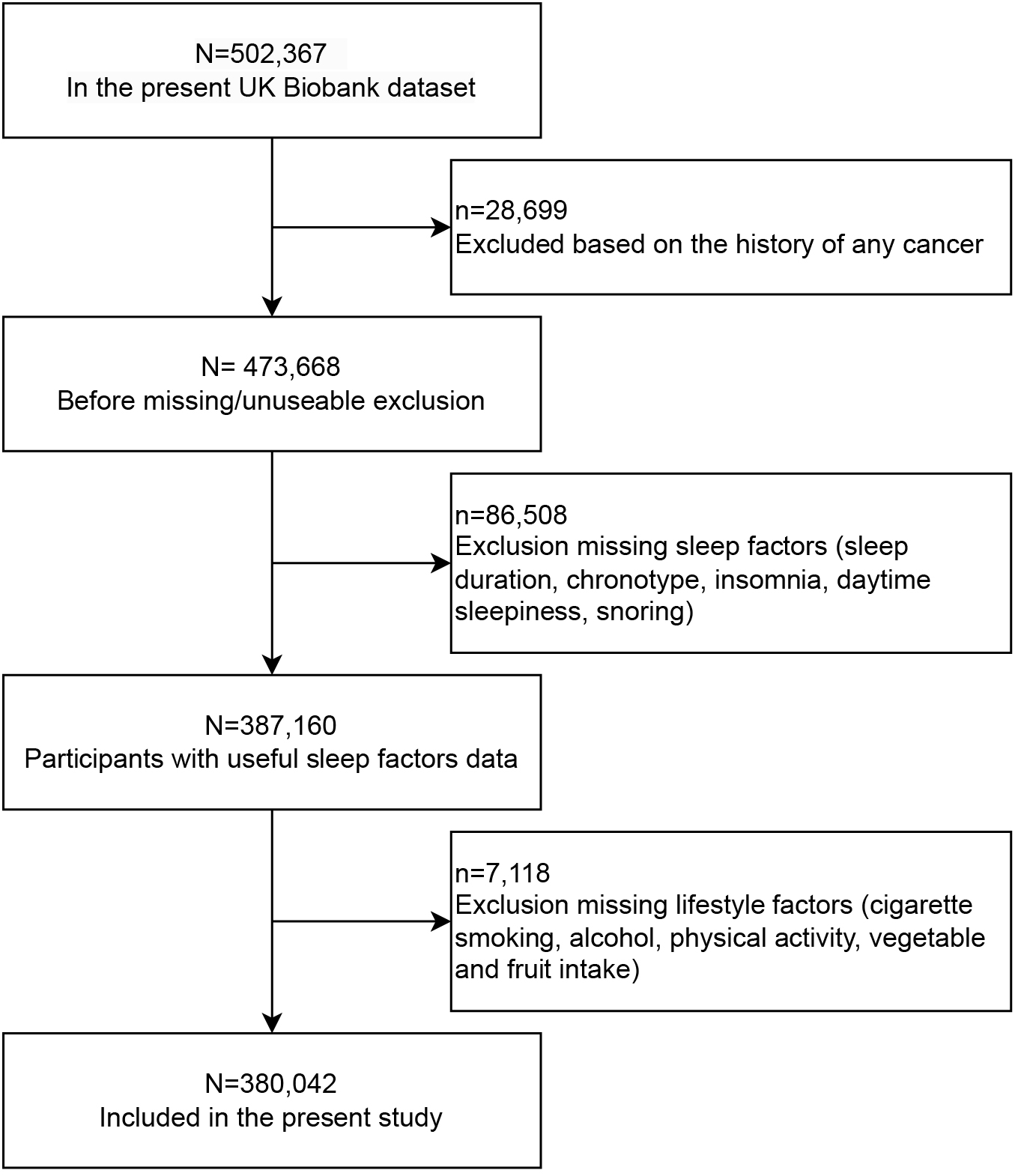
**

**Supplementary Figure S1. Flow chart of participants included in the study (United Kingdom, 2006–2016).**

**
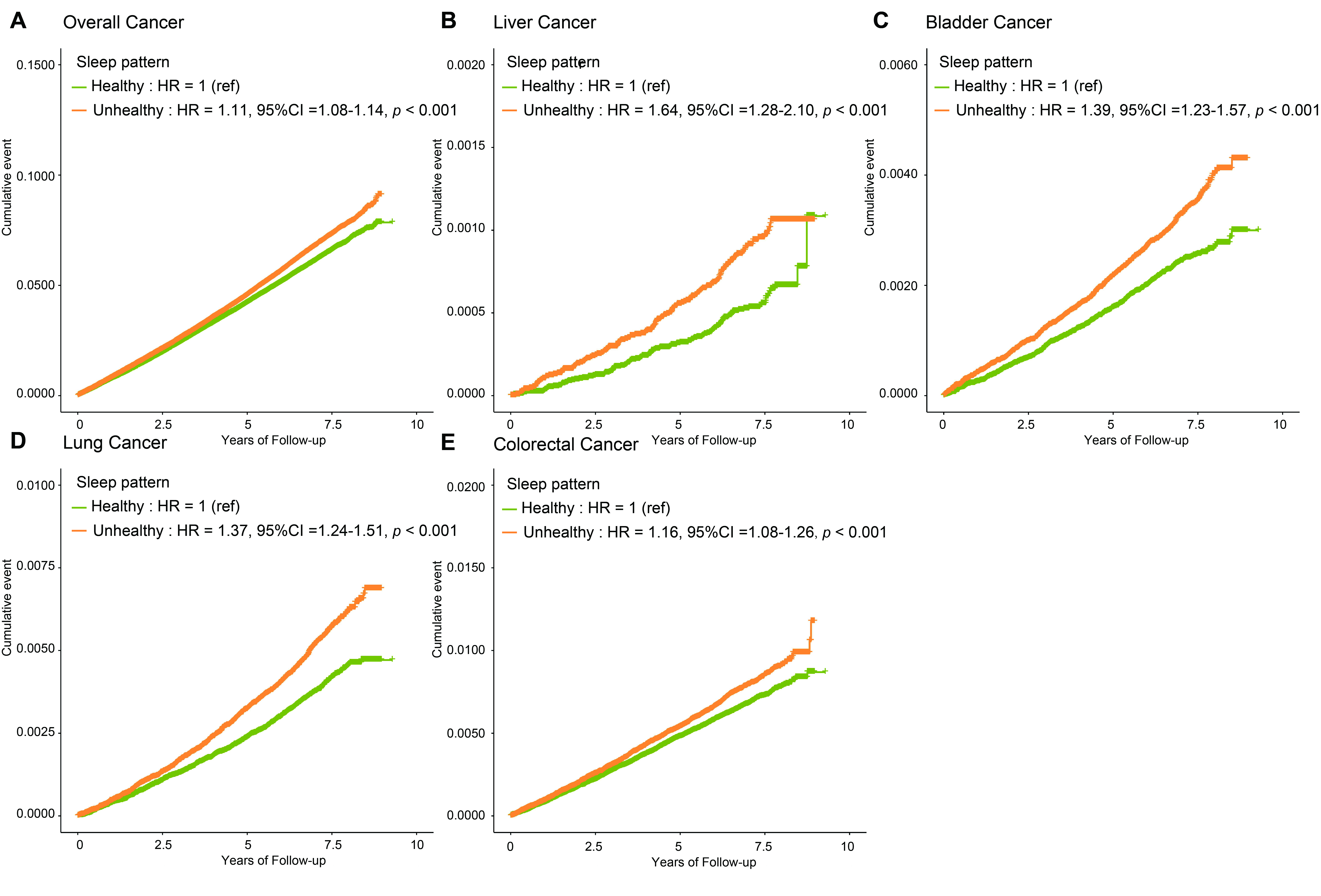
**

**Supplementary Figure S2. Cumulative incidences of overall cancer, liver cancer, bladder cancer, lung cancer, and colorectal cancer in the healthy and unhealthy sleep patterns (United Kingdom,2006–2016).** *Kaplan-Meier plot is used to present the results. The hazard ratios (HRs), corresponding 95% confidence intervals (CIs) and* P *values were calculated using the Cox regression model adjusting for sex, age, Townsend Deprivation Index, ethnicity, sedentary time, employment, education, BMI, and lifestyle pattern.*

**
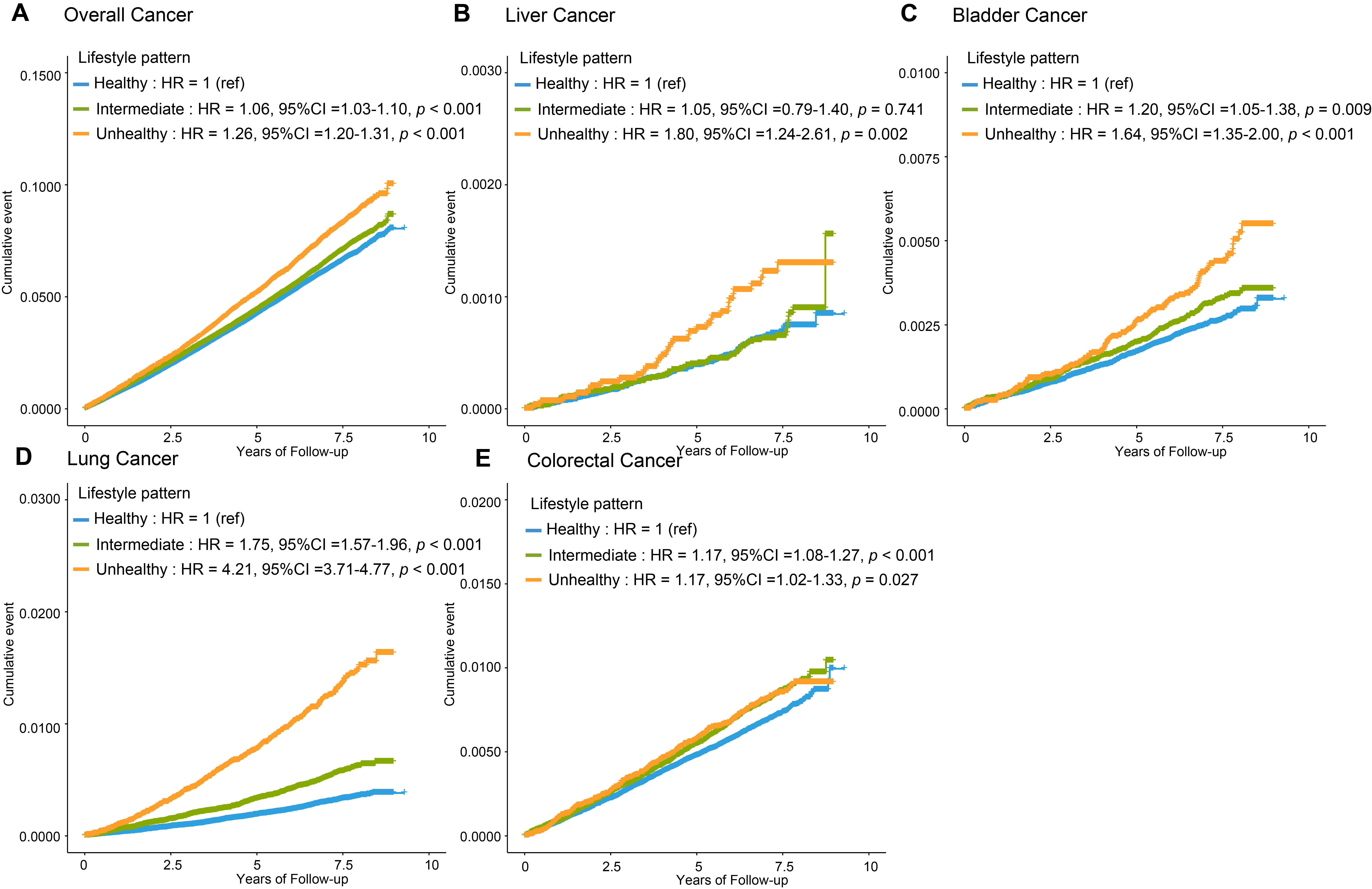
**

**Supplementary Figure S3. Cumulative incidences of overall cancer, liver cancer, bladder cancer, lung cancer, and colorectal cancer in the healthy, intermediate and unhealthy lifestyle patterns (United Kingdom,2006–2016).** *Kaplan-Meier plot is used to present the results. The hazard ratios (HRs), corresponding 95% confidence intervals (CIs) and* P *values were calculated using the Cox regression model adjusting for sex, age, Townsend Deprivation Index, ethnicity, sedentary time, employment, education, BMI, and sleep pattern.*

**
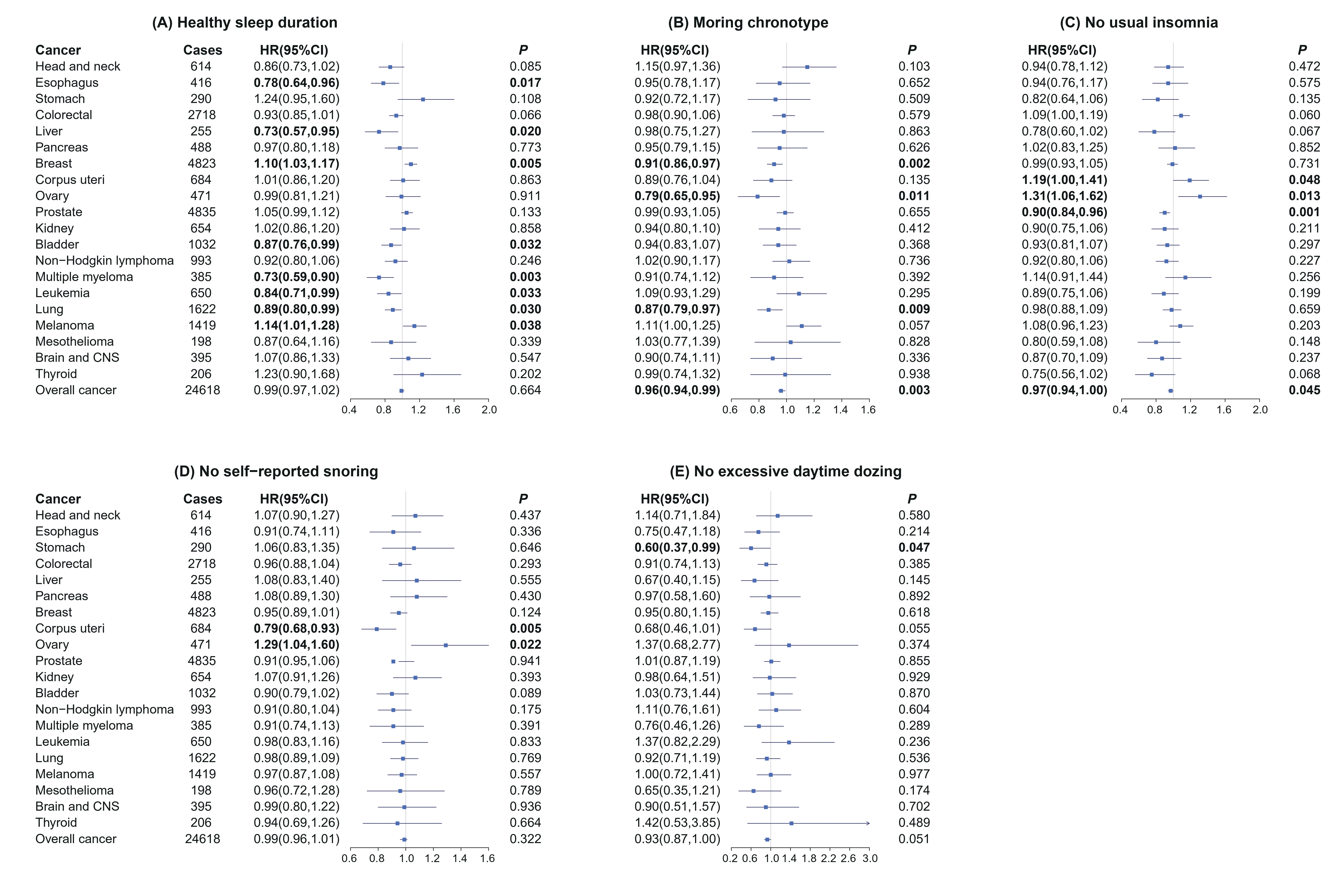
**

**Supplementary Figure S4. Associations of sleep factors with overall and 20 types of cancers (United Kingdom,2006–2016).** *Brain and CNS, brain and central nervous system cancers. The reference group was the group with the unhealthy sleep factor. Model was adjusted for sex, age, the Townsend Deprivation Index, ethnicity, sedentary time, employment, education, BMI, lifestyle pattern, and other sleep factors. Bold values provided* P *value <0.05.*

**
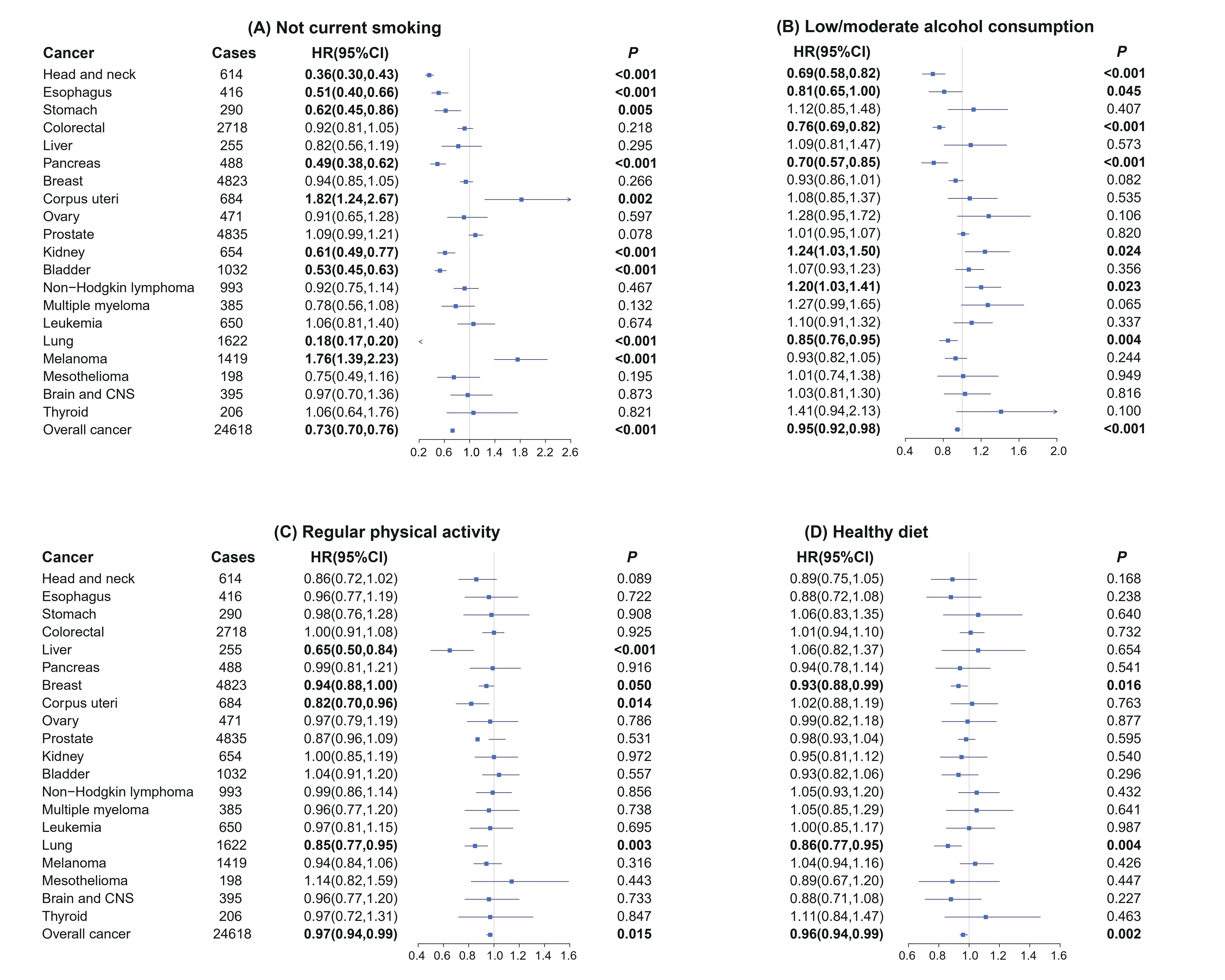
**

**Supplementary Figure S5. Associations of lifestyle factors with overall and 20 types of cancers (United Kingdom,2006–2016).** *Brain and CNS, brain and central nervous system cancers. The reference group was the group with the unhealthy lifestyle factor. Model was adjusted for sex, age, the Townsend Deprivation Index, ethnicity, sedentary time, employment, education, BMI, sleep pattern, and other lifestyle factors. Bold values provided* P *value <0.05.*
